# Tropomyosin-Related Kinase Receptor Type B Agonism in Geographic Atrophy—The Translational Challenges from Preclinical Data to a First-in-Human Trial

**DOI:** 10.1016/j.xops.2026.101216

**Published:** 2026-05-03

**Authors:** David Brown, Oliver Zeitz, Clare Bailey, Sunir Garg, Karl Csaky, James Talks, Sobha Sivaprasad, Peter M. Benz, Rolf Herrmann, Remko A. Bakker, Sebastian Bandholtz, Ankit Mittal, Qihong Huang, Serge Kosobokovs, Gudrun Simons, Andrea Giani, Jochen Huber, Martin Gliem, 1418.01 Study Group, Andrew Lotery, Sanjiv Banerjee, Tomas Burke, Nicholas Beare, Sergio Pagliarini, Brian B. Berger, Dennis Michael Marcus, William Zachery Bridges, David Stuart Boyer, Sunil S. Patel, John C. Randolph

**Affiliations:** 1 Retina Consultants of Texas, Retina Consultants of America, Houston, Texas; 2 Department of Ophthalmology, Charité-Universitätsmedizin Berlin, Berlin, Germany; 3 Bristol Eye Hospital, University Hospitals Bristol, Bristol Royal Infirmary NHS Trust, Bristol, UK; 4 Mid Atlantic Retina, The Retina Service of Wills Eye Hospital, Thomas Jefferson University, Philadelphia, Pennsylvania; 5 Retina Foundation of the Southwest, Dallas, Texas; 6 Royal Victoria Infirmary, Newcastle upon Tyne Hospitals NHS Foundation Trust, Newcastle Upon Tyne, UK; 7 National Institute for Health and Care Research (NIHR) Moorfields Biomedical Research Centre, Moorfields Eye Hospital, London, UK; 8 Boehringer Ingelheim Pharma GmbH & Co. KG, Biberach an der Riß, Germany; 9 C.H. Boehringer Sohn AG & Co. KG, Biberach an der Riß, Germany; 10 Boehringer Ingelheim Pharmaceuticals Inc., Ridgefield, Connecticut; 11 Boehringer Ingelheim International GmbH, Ingelheim am Rhein, Germany

**Keywords:** Age-related macular degeneration, Electroretinography, Ischemic optic neuropathy, Translational research, Tropomyosin-related receptor kinase B agonism

## Abstract

**Objective:**

To report results of the first-in-human trial of intravitreal tropomyosin-related kinase receptor type B (TrkB) agonist BI 754132 in participants with geographic atrophy (GA). To discuss BI 754132 clinical data in the context of the preclinical findings and their translation into human data.

**Design:**

An open-label, uncontrolled, nonrandomized phase I trial (NCT04002310) assessing the safety, tolerability, and pharmacokinetics of BI 754132 in participants with GA supported by preclinical data.

**Participants:**

Participants with GA recruited between July 2019 and August 2022 in the United States and UK.

**Methods:**

Clinical trial comprising a single rising dose (SRD) part (n = 15) and multiple dose (MD) part (n = 3). Of 18 participants treated, 16 received a single dose of BI 754132 0.3 to 6 mg (SRD, n = 15; MD, n = 1) and 2 received 3 doses each of BI 754132 6 mg (MD part).

**Main Outcome Measures:**

The primary SRD endpoint was the incidence of ocular and systemic dose-limiting events until day 100; the primary MD endpoint was treatment-related adverse events (AEs) until day 155. Exploratory endpoints included change from baseline in best-corrected visual acuity (BCVA), GA lesion area, central retinal thickness, and selected electroretinogram parameters.

**Results:**

Preclinical data supported a potential treatment effect and a favorable safety profile of BI 754132, supporting clinical development. In the phase I study (SRD and MD parts), 12 of 18 (67%) participants had an AE; of which, 4 had ischemic optic neuropathy in the study eye. Considering the frequency of ischemic optic neuropathy in the absence of efficacy data, the benefit–risk assessment of BI 754132 could not be considered as positive, and the clinical study was terminated. There was a mild increase in central retinal thickness (≤25 μm) of all study eyes. No relevant changes in BCVA, GA lesion area, or electroretinogram parameters were noted.

**Conclusions:**

Clinical data suggested a potential association between BI 754132 and development of ischemic optic neuropathy. Although the underlying mechanisms remain unclear, these data warrant caution during further exploration of TrkB agonism in GA and highlight the role of monitoring safety data during early clinical development, especially when studying new modes of action.

**Financial Disclosure(s):**

Proprietary or commercial disclosure may be found in the Footnotes and Disclosures at the end of this article.

Geographic atrophy (GA) is an advanced form of age-related macular degeneration (AMD) characterized by progressive atrophy of the retinal pigment epithelium (RPE), photoreceptor death, and vision loss.^1^, ^2^, ^3^, ^4^, ^5^, ^6^ As the prevalence of GA increases with age, the incidence of GA is projected to rise in countries with aging populations in the coming decades, representing a growing unmet need for new treatments.^7^ Geographic atrophy is a multifactorial disease with several avenues of investigation for treatment approaches, including synthetic vitamin A replacement therapy, visual cycle modulation, neuroprotection, RPE transplantation, gene therapy, stem cell therapy, and modulation of lipid metabolism.^8^, ^9^, ^10^, ^11^, ^12^, ^13^, ^14^

To date, most research studies have focused on blocking one of the underlying mechanisms of GA progression, complement activation.^15^ As of 2023, 2 pharmacological agents have been approved by the US Food and Drug Administration for the treatment of GA (pegcetacoplan and avacincaptad pegol), both of which are complement inhibitors.^16^^,^^17^ Both of these drugs require frequent (monthly or every other month) intravitreal administration, and their efficacy is limited, with limited impact on vision.^18^, ^19^, ^20^, ^21^ Additionally, in clinical trials, these agents were associated with an increased rate of neovascular AMD and intraocular inflammation.^16^, ^17^, ^18^ One potential explanation for the limited efficacy of current treatment options is the multifactorial pathogenesis of GA, including amyloid β or τ protein deposition, mitochondrial dysfunction, oxidative stress, dysfunction of extracellular matrix turnover, and iron overload.^22^, ^23^, ^24^, ^25^, ^26^, ^27^, ^28^, ^29^, ^30^ Accordingly, there is a need for additional treatment options for individuals with GA.

Effective neuroprotection is a therapeutic approach that could address multiple root causes of GA,^31^ and potentially achieve improved efficacy relative to or additive to complement inhibition. Neurotrophic factors regulate the development and function of the nervous system^32^ and have neuroprotective effects.^33^ The tropomyosin-related kinase receptor type B (TrkB) signaling pathway is well characterized in the visual system, particularly in relation to glaucoma, as well as retinal ganglion cell survival and axon regeneration.^34^, ^35^, ^36^ Tropomyosin-related kinase receptor type B and its ligand (brain-derived neurotrophic factor [BDNF]) are also expressed in human RPE cells^37^ involved in photoreceptor development.^38^ Individuals with AMD have notably lower BDNF levels compared with healthy individuals, and this reduction has been linked to a decrease in outer retinal layer thickness.^39^ Therefore, TrkB agonism may protect photoreceptors from degeneration, and counteract or delay vision loss in individuals with GA.^31^^,^^39^ Tropomyosin-related kinase receptor type B antibodies, which have a prolonged half-life relative to BDNF,^40^ may have greater therapeutic potential than the endogenous TrkB ligand.^31^

BI 754132 is an agonistic humanized immunoglobulin G1 antibody for TrkB, with a molecular weight of 146 kDa, designed to bind to the extracellular domain of human TrkB. Given the limited published data available on TrkB in GA, a series of *in vitro* and *in vivo* studies were conducted to establish the safety and preclinical efficacy of TrkB agonism for the treatment of GA. Here, we present data from the first-in-human phase I trial (NCT04002310) of the TrkB agonistic antibody BI 754132 in participants with GA in the context of preclinical data that formed the trial rationale.

## Preclinical Methodology

A TrkB agonistic tool antibody (C2) with high potency for mouse and rat TrkB (data on file) was used instead of BI 754132 when conducting studies in murine models. The *in vitro* cross-species reactivity of BI 754132 and the C2 tool antibody relative to that of BDNF was investigated in Chinese hamster ovary cells overexpressing TrkB (human, mouse, cynomolgus monkey, or rabbit) or tropomyosin-related receptor kinase A or C. The effects of TrkB agonism on intracellular signaling were investigated in human neuronal SH-SY5Y cells to provide insights into the mode of action of BI 754132 in humans.

The *in vivo* studies were conducted in murine models to investigate the effects of TrkB agonism (C2 tool antibody) on retinal function restoration and preservation, as well as retinal neuroprotection. Streptozotocin-induced diabetic rats with hyperglycemia-induced neuronal dysfunction in the retina, and mice with oxygen-induced retinopathy, were used to model retinal neurodegeneration and synaptic dysfunction. Subsequently, to aid in the selection of a suitable starting dose for the first-in-human clinical trial of BI 754132, the safety of intravitreal and intravenous BI 754132 was evaluated in cynomolgus monkeys. The full methods of all preclinical studies are detailed in the supplemental material, and their designs are summarized in Figures S1 and S2 (available at www.ophthalmologyscience.org).

## Preclinical Results

### *In Vitro* Characterization of BI 754132 and the C2 Tool Antibody

BI 754132 and the C2 tool antibody bound to the extracellular domain of human TrkB with an affinity of 1.4 and 8.4 nM, respectively, causing autophosphorylation and downstream signaling cascades (Table S1, available at www.ophthalmologyscience.org). BI 754132 and the C2 tool antibody acted as partial agonists for human and cynomolgus monkey TrkB (Tables S2 and S3, available at www.ophthalmologyscience.org). Unlike the endogenous TrkB agonist BDNF, which activated both tropomyosin-related receptor kinase A and C, BI 754132 and the C2 tool antibody were specific for TrkB (data not shown). Furthermore, BI 754132 and the C2 tool antibody were significantly more potent at TrkB phosphorylation than BDNF (Chinese hamster ovary cells, both *P* < 0.05; human neuronal SH-SY5Y cells, *P* < 0.01 [BI 754132] and *P* < 0.05 [C2 tool antibody]; Fig S3, available at www.ophthalmologyscience.org).

As expected, the immunoglobulin G1 anti-trinitrophenol (TNP) control antibody had no effect on TrkB phosphorylation. Consistently, the expression of genes regulating synaptic plasticity was increased in human neuronal SH-SY5Y cells stimulated with BDNF or BI 754118, a chimeric precursor of BI 754132 with a mixed mouse/human backbone, compared with control cells (Fig S4, available at www.ophthalmologyscience.org). Relative to control cells, both the C2 tool antibody and BI 754132 dose-dependently increased the amount of β3-tubulin staining (Fig S5, available at www.ophthalmologyscience.org) and reduced the levels of *NTRK2* mRNA that encodes the *TrkB* gene (Fig S4).

### Effects of the C2 Tool Antibody on Retinal Function in Murine Models

Intravitreal injections of the C2 tool antibody and the anti-TNP control antibody were generally well tolerated in murine models, with no overt tolerability issues.

Blood glucose levels of streptozotocin-induced diabetic control rats following insulin treatment were compared with those of nondiabetic control rats (approximately 5 mM). As expected, the streptozotocin-induced diabetic rats administered the anti-TNP control antibody (diabetic control group) displayed progressive loss of retinal function. Blood glucose levels of streptozotocin-induced diabetic rats were not affected by administration of the C2 tool antibody and anti-TNP control antibody. The C2 tool antibody (0.01–50 μg) did, however, improve retinal function in a dose and time-dependent manner, with the highest tested dose (50 μg) achieving the longest-lasting protective effect. Two intravitreal injections of 50 μg of the C2 tool antibody completely preserved normal light sensitivity, saturating b-wave response amplitude, photopic negative response, and outer retinal contrast sensitivity, whereas insulin treatment preserved only the photopic negative response (partially) and contrast sensitivity (fully; Fig S6, available at www.ophthalmologyscience.org). A single intravitreal injection of 50 μg of the C2 tool antibody significantly improved light sensitivity (rod driven, *P* < 0.01; UV-cone–driven, *P* < 0.05), saturating b-wave response amplitude (rod driven, *P* < 0.01; M-cone–driven, *P* < 0.001), and photopic negative response (*P* < 0.05), and completely restored the b-wave implicit time (*P* < 0.001); however, it did not improve a-wave responses (Fig S7, available at www.ophthalmologyscience.org).

Likewise, in the oxygen-induced retinopathy mouse model, the improvements in rod-driven and M-cone–driven b-wave light sensitivity and pattern electroretinography (ERG) responses observed following the second injection of 11 μg of the C2 tool antibody were greater than those reported after the first injection (Fig S8, available at www.ophthalmologyscience.org). However, although C2 tool antibody treatment increased the number of vital ganglion cells to the level observed in normoxic controls (Fig S9, available at www.ophthalmologyscience.org), it did not affect the retinal layer thickness or the retinal vasculature. The retinal function impairments of control oxygen-induced retinopathy mice did not recover during the study.

### Safety and Toxicokinetics of BI 754132 in Cynomolgus Monkeys

#### Cynomolgus Monkey Intravenous Injection Study

BI 754132 was well tolerated when administered at 3, 10, or 50 mg/kg doses via intravenous injection once weekly for 13 weeks to cynomolgus monkeys. BI 754132-related increases in bodyweight of monkeys administered BI 754132 at 3 and 10 mg/kg doses were consistent with the bodyweight changes previously reported for the C2 tool antibody in nonhuman primates.^41^ As no adverse effects were reported in the study, the no observed adverse effect level (NOAEL) was established to be 50 mg/kg, corresponding to mean maximum serum concentration of BI 754132 after a single intravitreal dose (C_max_) and area under the concentration–time curve of BI 754132 in serum over the time interval from 0 to 168 h values of 2270 μg/mL and 252 000 μgċh/mL at the end of the study, respectively (Table S4, available at www.ophthalmologyscience.org).

#### Cynomolgus Monkey Intravitreal Injection Studies

Overall, multiple intravitreal BI 754132 doses were well tolerated in cynomolgus monkeys. Following intravitreal administration of BI 754132 (1, 3, or 6 mg/eye) or vehicle control to the right eye of cynomolgus monkeys once every 4 weeks for 13 weeks (4 doses in total), immunogenicity-related intraocular inflammation was observed in 3 female monkeys (2/6 [33%] in 1 mg/eye dose group and 1/6 [17%] in 3 mg/eye dose group) and correlated with high antidrug antibody signals in these animals. This degree of inflammation can, in general, be expected with humanized antibody drugs when injected in nonhuman primates. However, no meaningful BI 754132-related direct effects were observed on ophthalmic examination, ocular photography, ERG, or histopathology of the eye. Thus, the NOAEL for the 13-week monkey intravitreal study was 6 mg/eye, excluding the immunogenicity-related inflammatory responses in the 3 animals in the 1 and 3 mg/eye dose groups. This dose corresponded to C_max_ and area under the concentration–time curve of BI 754132 in serum over the time interval from 0 to 672 hours, with values of 9.88 μg/mL and 2010 μgċh/mL, respectively, in male monkeys and 12.10 μg/mL and 2110 μgċh/mL, respectively, in female monkeys on day 57.

Intraocular inflammation was also reported in a 26-week study of intravitreal BI 754132 (1, 3, or 6 mg/eye). Following administration of BI 754132 or vehicle control to the right eye of cynomolgus monkeys once every 4 weeks for 26 weeks (7 doses in total), intraocular inflammation was observed in 9 monkeys (5/8 [63%] in the 1 mg/eye dose group, 2/8 [25%] in the 3 mg/eye dose group, and 2/8 [25%] in the 6 mg/eye dose group). As in the 13-week study, intraocular inflammation was correlated with high antidrug antibody signals, and no BI 754132-related changes were observed on ophthalmic examination or ERG. Quantitative OCT evaluation revealed mild retinal nerve fiber layer (RNFL) and retinal thickening at weeks 4, 13, and 26 of the dosing phase in some eyes administered BI 754132. Although there was no clear dose response, RNFL thickening was observed in more eyes at weeks 13 and 26 relative to week 4. The NOAEL for the 26-week monkey intravitreal study was established as 6 mg/eye.

#### Human Starting Dose Calculation

The vitreous volume of human eyes is approximately 2 times larger than that of cynomolgus monkey eyes^42^; therefore, the 6 mg/eye NOAEL established in cynomolgus monkeys was extrapolated to be equivalent to 12 mg/eye in humans. A safety factor of 10 was implemented to account for extrapolation from animal to human, and an additional fourfold factor was applied to account for the difference in *in vitro* functional activity of BI 754132 in cynomolgus monkeys and humans. With consideration of these safety factors, 0.3 mg/eye was determined as a safe starting dose for the first-in-human study of BI 754132.

## Clinical Methodology

### Trial Design and Participants

**Figure 1:**
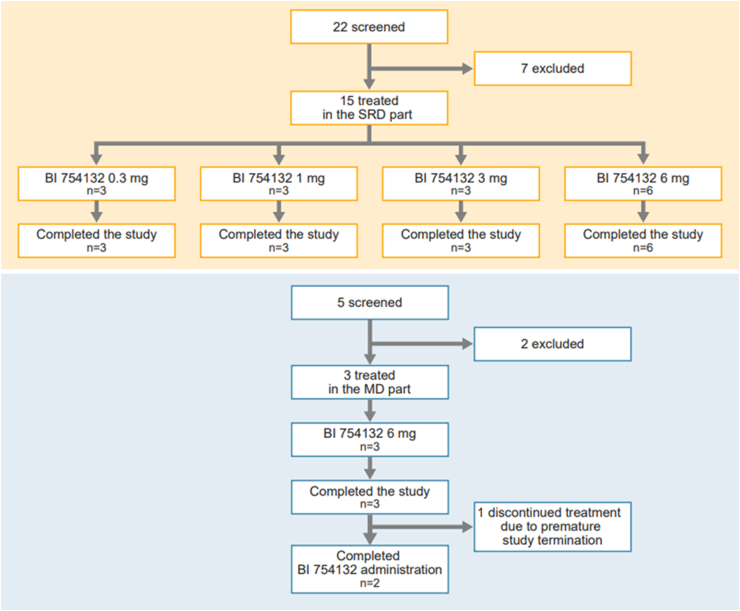
CONSORT flow diagram for the phase I study (SRD part, orange; MD part, blue). MD = multiple dose; SRD = single rising dose.

Based on toxicology data and a promising mechanism of action established in preclinical models, BI 754132 entered clinical development. The first-in-human, multicenter, open-label, uncontrolled, and nonrandomized phase I trial (NCT04002310) examined the safety, tolerability, and pharmacokinetics (PK) of intravitreal BI 754132 in participants with GA secondary to AMD recruited in the United States and UK. The study comprised a single rising dose (SRD) part followed by a multiple dose (MD) part (Fig 1). In the SRD part, participants were divided into 4 dose groups to receive a single dose of intravitreal BI 754132 (0.3, 1, 3, or 6 mg). Participants received BI 754132 on day 1 and were subsequently followed up for 100 days. In the MD part, participants received 3 doses of BI 754132 6 mg administered every 4 weeks and were followed up for up to 155 days. Randomization and masking information is provided in the Supplemental material (available at www.ophthalmologyscience.org).

Participants were eligible for the SRD part if they were adults aged ≥50 years with GA secondary to AMD, a GA lesion of at least 1.9 mm^2^ disc area in size and a best-corrected visual acuity (BCVA) of 20/100–20/400 (corresponding to 19–53 letters on the ETDRS chart; measured by the ETDRS protocol). Participants eligible for the MD part had a GA lesion of at least 7.5 mm^2^ disc area in size and a BCVA of 20/100 or lower (<53 letters on the ETDRS chart). The full list of selection criteria is provided in Table S5 (available at www.ophthalmologyscience.org). Further enrollment of patients into the trial was to be interrupted if: A drug-related serious adverse event (AE) was reported (both study parts) or if ischemic optic neuropathy or relevant predisposing changes to the optic disc were seen in the study eye after administration of BI 754132 (MD part only).

The study protocol was reviewed and approved by The Wills Eye Hospital Institutional Review Board, the Medicines and Healthcare products Regulatory Agency, and the US Food and Drug Administration. All participants provided written informed consent before entering the trial; the trial was conducted in accordance with Good Clinical Practice, the ethical principles laid down in the Declaration of Helsinki, the European Union medical devices directive (93/42/EEC), the European Union directive 2001/20/EC regulation number 536/2014, the harmonized standards for medical devices (ISO 14155), and other applicable regulatory requirements. A safety monitoring committee regularly reviewed safety data and made decisions on dose escalation and continuation of the trial.

### Dose Selection and Administration

Selection for the SRD part was based on the preclinical data in cynomolgus monkeys. A Bayesian logistic regression model, based on a weakly informative prior distribution and employing the escalation with overdose control principle, was used to guide dose escalation; doses of BI 754132 0.3, 1, 3, and 6 mg/eye were selected. Dose selection for the MD part was based on the highest tolerable dose (6 mg) from the SRD part. BI 754132 was administered to the study eye as a single intravitreal injection; participants in the SRD part received a single dose of BI 754132 0.3–6 mg, and participants in the MD part received 3 doses of BI 754132 6 mg, administered 4 weeks apart. The proposed starting dose of 0.3 mg/eye was predicted to maintain the minimal efficacious intravitreal concentration of 6.38 nM for 60 days, using preclinical data (Table S3).

### Study Outcomes

#### Single Rising Dose Part

The primary endpoint was the number of participants with ocular (in the study eye) and systemic dose-limiting events from first treatment administration until the end of the study (day 100). Dose-limiting events were defined as development of sterile endophthalmitis and/or sterile inflammation of the vitreous of grade 3 or higher for at least 5 days (according to Standardization of Uveitis Nomenclature working group grading scheme);^43^ intraocular pressure (IOP) of at least 30 mmHg for 3 days; signs of vascular occlusion in the retina (including peripheral retinal hemorrhage, but not hemorrhage of the macula); or visual acuity loss of more than 15 letters in the study eye between any 2 consecutive visits. Secondary endpoints were the number of participants with treatment-related AEs or ocular AEs (eye disorders) in the study eye, as well as selected PK parameters (including C_max_, area under the concentration–time curve of BI 754132 in serum over the time interval from 0 extrapolated to infinity and time from dosing to maximum serum concentration of BI 754132).

#### Multiple Dose Part

The primary endpoint was the number of participants with treatment-related AEs from first treatment administration until the end of the study (day 155). Secondary endpoints were the trough levels of BI 754132 before administration of the second and third doses, and the plasma BI 754132 concentration 4, 8, and 14 weeks after administration of the last dose. Systemic accumulation of BI 754132 after the third dose was also measured.

#### Both Study Parts

Additional endpoints of both study parts included ophthalmic parameters, including changes from baseline in BCVA, GA lesion area (based on fundus autofluorescence imaging), central retinal thickness, and total macular volume (based on spectral-domain OCT imaging and ERG b-wave implicit times and amplitudes recorded under photopic conditions), for each time point in the study and fellow eyes.

### Ophthalmic Assessments

All ophthalmic examinations were performed in both eyes. Electroretinography recordings, BCVA assessment, ocular tonometry for IOP determination, and slit-lamp examinations (all visits) were performed before pupil dilation. Further ophthalmic examinations performed after pupil dilation included indirect ophthalmoscopy (MD part only; all visits), color fundus photography, fundus autofluorescence (Heidelberg Spectralis, Heidelberg Engineering), and spectral-domain OCT (Heidelberg Spectralis, Heidelberg Engineering). Ophthalmic images were graded by an independent central reading center.

### Further Assessments

Further safety assessments included physical examinations (vital signs), safety laboratory assessments, and 12-lead electrocardiograms.

### Statistical Analysis

No confirmatory hypothesis testing was planned; descriptive statistics were calculated for safety and PK parameters. Participants were analyzed according to the treatment assigned to them at the start of the study. Primary, secondary, and further endpoints were evaluated using the treated set, which included all participants who received at least 1 dose of the trial treatment. Pharmacokinetics parameter analyses included all participants in the treated set who had at least 1 measurable PK endpoint. If no dose-limiting events occurred, enrollment of 15 participants in the SRD part was deemed sufficient for the investigation of the trial endpoints. No formal interim analyses were planned or performed.

## Clinical Results

### Study Participants

Between July 2019 and August 2022, 18 participants were included in the trial (Fig 1). In the SRD part, 15 participants received a single dose of BI 754132 (0.3, 1, and 3 mg, n = 3 each; 6 mg, n = 6). Of the 3 participants included in the MD part, 2 received 3 doses of BI 754132 6 mg and one received a single dose of BI 754132 6 mg prior to trial termination. Baseline characteristics were similar across the study parts; all participants were White, most were female (SRD part, 10/15 [66.7%]; MD part, 2/3 [66.7%]), and the mean age was 77 years (Table 1).

**Table 1:** Participant Baseline Characteristics (TS)

Characteristic	SRD Part				SRD Part Total (N = 15)	MD Part
	BI 754132 0.3 mg (n = 3)	BI 754132 1 mg (n = 3)	BI 754132 3 mg (n = 3)	BI 754132 6 mg (n = 6)		BI 754132 6 mg (N = 3)
Sex, n (%)						
Male	1 (33.3)	2 (66.7)	1 (33.3)	1 (16.7)	5 (33.3)	1 (33.3)
Female	2 (66.7)	1 (33.3)	2 (66.7)	5 (83.3)	10 (66.7)	2 (66.7)
Mean age (SD), yrs	78.3 (4.9)	78.7 (2.5)	70.7 (8.1)	77.7 (6.0)	76.6 (6.0)	77.3 (8.3)
Mean BMI (SD), kg/m^2^	27.3 (5.2)	30.9 (2.9)	37.7 (12.1)	27.6 (3.3)	30.2 (6.9)	25.2 (5.2)
Race, n (%)						
White∗	3 (100)	3 (100)	3 (100)	6 (100)	15 (100)	3 (100)

BMI = body mass index; MD = multiple dose; SD = standard deviation; SRD = single rising dose; TS = treated set.

∗ Non-Hispanic/Latino.

### Safety Outcomes

#### SRD Part

No dose-limiting events were detected. In total, 9 of 15 (60%) participants were reported as having an AE. Three participants (3/15 [20.0%]) experienced events, which were considered by the investigator to be related to treatment: Ocular procedural complication, n = 1; photopsia and vitreous opacities, n = 1; and visual impairment, n = 1 (Table 2). Overall, 6 of 15 (40.0%) participants had ocular events in the study eye (Table 3), including 2 participants who were reported as having a serious AE of ischemic optic neuropathy of moderate intensity.

**Table 2:** Summary of BI 754132 Safety (TS)

Proportion of Participants with an AE, n (%)	SRD Part				SRD Part Total (N = 15)	MD Part
	BI 754132 0.3 mg (n = 3)	BI 754132 1 mg (n = 3)	BI 754132 3 mg (n = 3)	BI 754132 6 mg (n = 6)		BI 754132 6 mg (N = 3)
Any AE	1 (33.3)	1 (33.3)	3 (100)	4 (66.7)	9 (60.0)	3 (100)
Ocular	1 (33.3)	1 (33.3)	2 (66.7)	2 (33.3)	6 (40.0)	3 (100)
In the study eye	1 (33.3)	1 (33.3)	2 (66.7)	2 (33.3)	6 (40.0)	NR
Serious	1 (33.3)	0	0	1 (16.7)	2 (13.3)	2 (66.7)
Ischemic optic neuropathy	1 (33.3)	0	0	1 (16.7)	2 (13.3)	2 (66.7)∗
Procedure-related	1 (33.3)	1 (33.3)	1 (33.3)	1 (16.7)	4 (26.7)	0
AE of special interest	0	0	0	0	0	1 (33.3)∗
Investigator-defined, treatment-related	1 (33.3)	0	1 (33.3)	1 (16.7)	3 (20.0)	2 (66.7)
Ischemic optic neuropathy	0	0	0	0	0	2 (66.7)†
Photopsia	0	0	1 (33.3)	0	1 (6.7)	0
Visual impairment	0	0	0	1 (16.7)	1 (6.7)	0
Vitreous opacities/floaters	0	0	1 (33.3)	0	1 (6.7)	0
Ocular procedural complication	1 (33.3)	0	0	0	1 (6.7)	0
Serious	0	0	0	0	0	2 (66.7)

AE = adverse event; MD = multiple dose; NR = not reported; SRD = single rising dose; TS = treated set.

∗ One participant was reported to have eye hemorrhage and papilledema, which were AEs of special interest; this case was later redefined as an ischemic optic neuropathy case (represented in both categories in this table).

† The second event was reported after trial termination and was originally reported as eye hemorrhage and papilledema.

**Table 3:** Summary of Ocular Events (TS)

Proportion of Participants with an Ocular AE, n (%)	SRD Part				SRD Part Total (N = 15)	MD Part
	BI 754132 0.3 mg (n = 3)	BI 754132 1 mg (n = 3)	BI 754132 3 mg (n = 3)	BI 754132 6 mg (n = 6)		BI 754132 6 mg (N = 3)
Any ocular AE	1 (33.3)	1 (33.3)	2 (66.7)	2 (33.3)	6 (40.0)	3 (100)
In the study eye	1 (33.3)	1 (33.3)	2 (66.7)	2 (33.3)	6 (40.0)	NR
Ischemic optic neuropathy	1 (33.3)	0	0	1 (16.7)	2 (13.3)	2 (66.7)∗
Conjunctival hemorrhage	0	1 (33.3)	0	0	1 (6.7)	0
Retinal hemorrhage	0	0	0	0	0	1 (33.3)
Dry eye	0	0	0	1 (16.7)	1 (6.7)	0
Eye pruritus	0	0	1 (33.3)	0	1 (6.7)	0
Lacrimation increased	0	0	1 (33.3)	0	1 (6.7)	0
Photophobia	0	0	0	1 (16.7)	1 (6.7)	0
Photopsia	0	0	1 (33.3)	0	1 (6.7)	0
Visual impairment	0	1 (33.3)	0	1 (16.7)	2 (13.3)	0
Vitreous opacities/floaters	0	0	1 (33.3)	0	1 (6.7)	1 (33.3)
Ocular procedural complication	1 (33.3)	0	0	0	1 (6.7)	0

AE = adverse event; MD = multiple dose; NR = not reported; SRD = single rising dose; TS = treated set.

∗ The second event was reported after trial termination and was originally reported as eye hemorrhage and papilledema.

**Figure 2:**
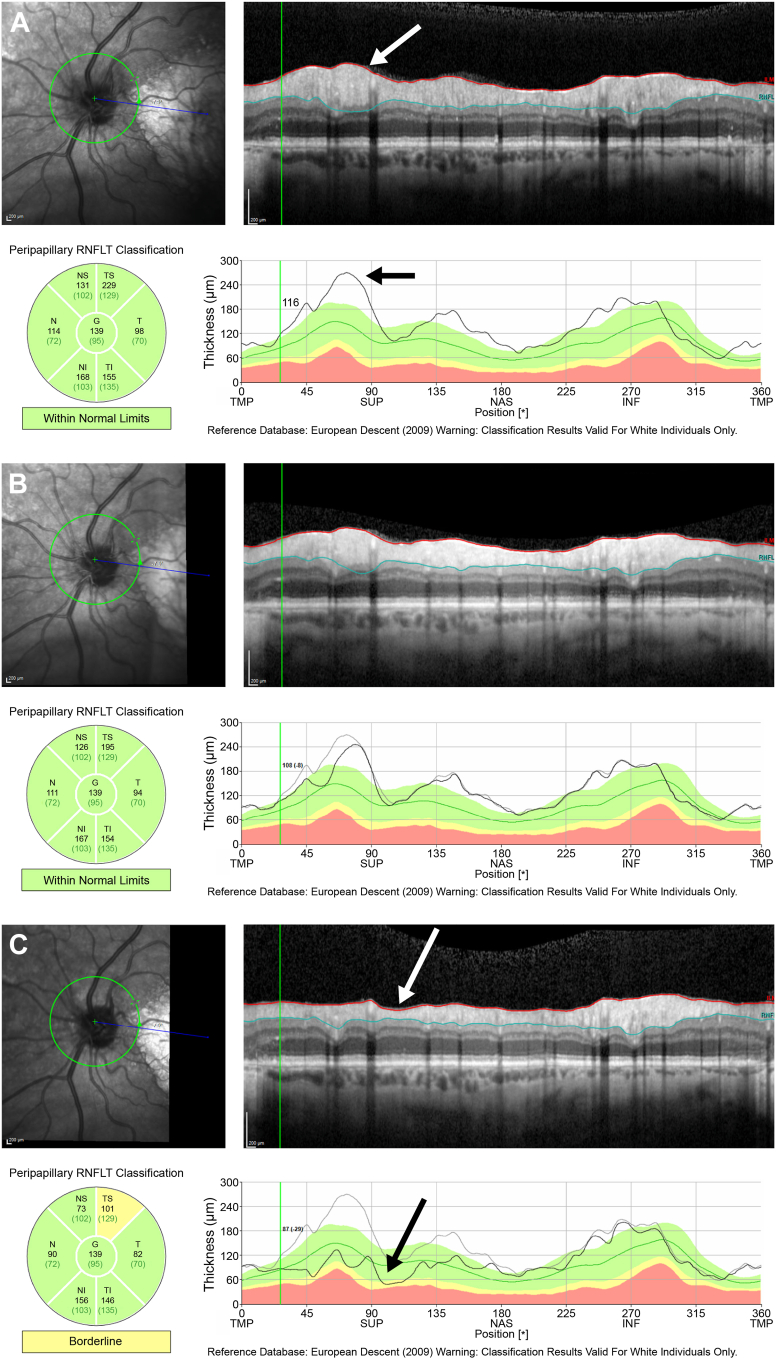
Spectral-domain OCT images of the optic nerve head of a 76-year-old participant with ischemic optic neuropathy at the time of diagnosis (84 days after BI 754132 administration **[A]**), after 7 days of follow-up **(B)** and after 6 weeks of follow-up **(C)**. The arrows in panel A mark the area of increased retinal thickness on SD-OCT and compared with normative data; the arrows in panel C mark the corresponding atrophy after ischemic optic neuropathy resolution. G = global; INF = inferior; N = nasal; NAS = nasal; NI = nasal inferior; NS = nasal superior; RNFLT = retinal nerve fiber layer thickness; SD-OCT = spectral-domain OCT; SUP = superior; T = temporal; TI = temporal inferior; TMP = temporal; TS = temporal superior.

The first case of ischemic optic neuropathy developed in a 76-year-old asymptomatic male participant and was discovered during a planned, routine clinical examination when slight superior sectorial optic nerve head swelling was noted. The participant had received one 0.3 mg dose of BI 754132 84 days previously. Spectral-domain OCT revealed increased thickness of the RNFL in the superior quadrant (Fig 2A). Best-corrected visual acuity was stable, and there were no visual field defects measurable. The participant had pre-existing hyperlipidemia and a relatively small optic disc. During follow-up, the RNFL swelling gradually decreased after 7 days (Fig 2B) and resulted in a small RNFL defect after resolution, 6 weeks after the initial presentation (Fig 2C).

**Fig 3:**
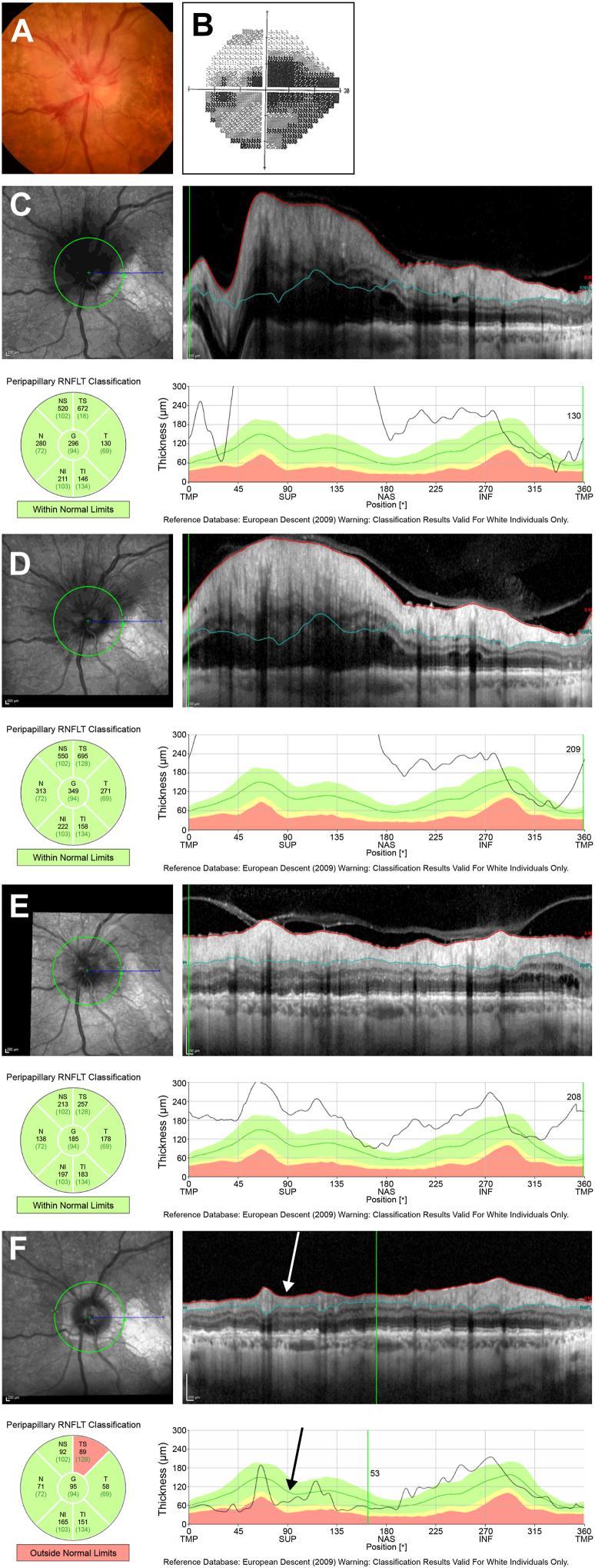
Fundus color image **(A)**, a standard automated perimetry image **(B)** and an SD-OCT image of the optic nerve head **(C)** of a 72-year-old participant with ischemic optic neuropathy at the time of diagnosis (44 days after BI 754132 administration). Panels **D–F** represent SD-OCT follow-up images taken 7 days **(D)**, 6 weeks **(E)**, and 12 weeks **(F)** after initial diagnosis. The arrows in panel F mark the area of atrophy on SD-OCT and compared with normative data. G = global; INF = inferior; N = nasal; NAS = nasal; NI = nasal inferior; NS = nasal superior; RNFLT = retinal nerve fiber layer thickness; SD-OCT = spectral-domain OCT; SUP = superior; T = temporal; TI = temporal inferior; TMP = temporal; TS = temporal superior.

The second case occurred in a 72-year-old male participant, with pre-existing hypertension, 44 days after injection of BI 754132 6 mg. The participant noted a visual field defect and blurred vision for approximately 1 week, and examination revealed swelling and hemorrhages of the optic disc (predominantly in the superior and temporal quadrant; Fig 3A). Spectral-domain OCT showed corresponding swelling of retinal layers (Fig 3B). Visual field testing demonstrated a central defect from the GA and diffuse loss of sensitivity in the inferior central visual field (Fig 3C). The participant was diagnosed with an ischemic optic neuropathy and scheduled for follow-up examinations. During follow-up, there was a gradual improvement of the retinal thickening over time, resulting in an RNFL defect after resolution of edema at week 12 (Fig 3D–F).

#### MD Part

Adverse events and ocular AEs in the study eye reported during the MD part are summarized in Tables 2 and 3, respectively. Two participants (2/3; 66.7%) were considered by the investigator to have an AE related to treatment.

A third case of ischemic optic neuropathy of moderate intensity was reported in a 69-year-old female participant, 42 days after the administration of the third dose of BI 754132 6 mg. Similar to the second case, the participant reported a new-onset visual field defect. On examination, superotemporal swelling and hemorrhages of the optic nerve head were identified, with corresponding swelling of the retinal layers on OCT and an inferior defect on vision field testing. This case of ischemic optic neuropathy was then considered by the investigator to be serious and related to the study drug. The study was terminated and no further doses of BI 754132 were administered because the benefit–risk balance of BI 754132 was no longer considered acceptable. After the trial was stopped, another case of ischemic optic neuropathy was reported in another participant, 108 days after the administration of a single 6 mg dose of BI 754132. The event was of moderate intensity and reported as serious and drug-related.

### Ophthalmic Outcomes

**Fig 4:**
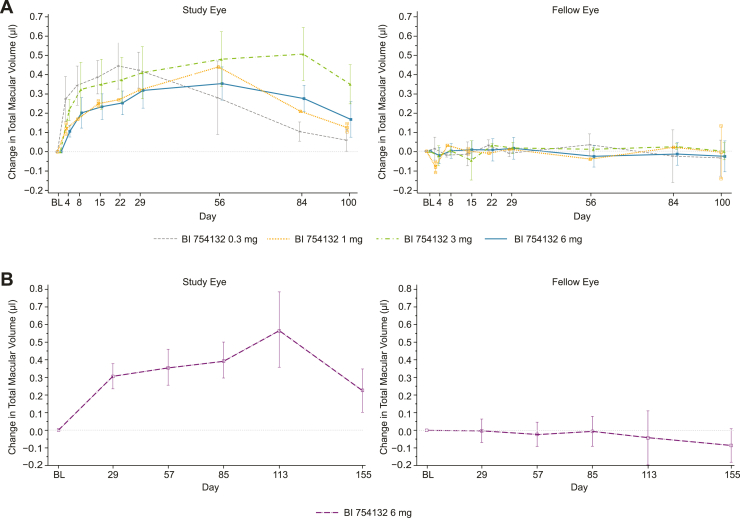
Mean change from baseline in total macular volume of the study and fellow eyes over time in the SRD **(A)** and MD **(B)** parts of the phase I trial (TS). Error bars show SD. Baseline was defined as treatment visit 2 (day 1). If no BL value was available at visit 2, BL was defined as the last measurement taken at screening (visit 1; day –3). BL = baseline; MD = multiple dose; SD = standard deviation; SRD = single rising dose; TS = treated set.

Over the course of the study, there were no relevant mean changes noted in BCVA or GA lesion size in study eyes or fellow eyes, nor were there any observed differences between the dose groups (Figs S10 and S11, available at www.ophthalmologyscience.org). Small but consistent mean increases from baseline in total macular volume (up to ∼0.56 μL, Fig 4) and central retinal thickness (up to 14 μm; Fig S12, available at www.ophthalmologyscience.org) were observed in all study eyes throughout the study. The greatest mean increase in total macular volume was reached within 29–84 days of BI 754132 administration, with higher BI 754132 doses resulting in slightly longer-lasting effects. Notably, the exclusion of total macular volume measurements obtained after occurrence of ischemic optic neuropathy did not appreciably change these results (data not shown). The total macular volume and central retinal thickness of fellow eyes remained mostly stable during the same time period (Fig 4 and S12).

BI 754132 had no clear effect on b-wave implicit times in humans. Except for the BI 754132 3 mg group, for which b-wave implicit time was higher at 800 Td in the study eyes than in the fellow eyes throughout the follow-up, there were no clear differences in b-wave implicit time of the study and fellow eyes for up to 100 days of follow-up in the SRD part (Fig S13, available at www.ophthalmologyscience.org). Similarly, following administration in the MD part, the b-wave implicit time of fellow and study eyes was similar during the 155 days of follow-up (Fig S14, available at www.ophthalmologyscience.org).

### PK Outcomes

Pharmacokinetic assessments were limited by the small number of participants with measurable concentrations of BI 754132 in the plasma. Pharmacokinetic parameters were calculable for 2 participants in the SRD part; none of the participants in the MD part had measurable serum concentrations of BI 754132. Therefore, descriptive statistics could not be calculated for PK outcomes in either of the study parts.

## Discussion

As TrkB plays a key role in photoreceptor development, activity, and survival,^38^^,^^44^ stimulation of TrkB signaling could potentially protect photoreceptors and RPE cells from degeneration, counteracting, or delaying vision loss in people with GA. BI 754132 is an agonistic humanized immunoglobulin G1 antibody for TrkB, with high selectivity and specificity for human and cynomolgus monkey TrkB, which showed favorable safety and early efficacy in preclinical models. However, intravitreal application of BI 754132 demonstrated a considerably higher frequency of ischemic optic neuropathy occurrence compared with available epidemiological data.^45^

Ischemic optic neuropathy is a serious condition that may result in irreversible loss of vision. There are no therapies with proven efficacy for ischemic optic neuropathy,^46^^,^^47^ although there are data from uncontrolled studies and case series suggesting potential benefits with steroids, if initiated early.^45^^,^^48^ Presentation is typically sudden, painless and comprises variable vision loss accompanied by swelling of the optic disc with or without hemorrhages.^46^ Prognosis is variable,^46^ with some improvement over time; however, complete recovery from ischemic optic neuropathy is uncommon.^48^ The pathophysiology is not completely understood but includes insufficient blood flow within the capillaries supplying the optic nerve head and varies based on individual predisposition.^46^ Predisposition includes smaller optic disc size, increased IOP, cardiovascular risk factors, and sleep apnea.^47^

Review of the 4 ischemic optic neuropathy cases reported in this trial revealed some common features. All 4 cases occurred in the study eye, relatively late after intravitreal injection of BI 754132 (44–108 days). Symptoms varied from mild reductions in BCVA and mild-to-moderate visual field defects to asymptomatic cases. Indirect ophthalmoscopy revealed swelling and hemorrhages of the optic nerve at the time of examination. The changes ranged from mild sectoral swelling to swelling affecting most of the optic disc. OCT RNFL scanning revealed reflectivity changes of the outer retinal layers and swelling. Follow-up data showed a gradual decrease in RNFL thickening. At least 2 participants had evidence of sectoral atrophic thinning after ischemic optic neuropathy, suggesting atrophic changes. Overall, the presentation of ischemic optic neuropathy cases described above resembled that of nonarteritic anterior ischemic optic neuropathy; none of the 4 cases observed in this trial had evidence of temporal arteritis associated with arteritic anterior ischemic optic neuropathy.

Nonarteritic anterior ischemic optic neuropathy is the most common form of ischemic optic neuropathy in older adults, with an estimated annual incidence of 2.3–10.2 per 100 000 individuals in adults aged >50 years in the United States.^45^ This incidence rate contrasts clearly with the incidence of over 20% reported in this trial. A causal relationship between BI 754132 and ischemic optic neuropathy was considered likely, as all of the cases of ischemic optic neuropathy presented in the study eye had similar clinical features and occurred at a higher rate than expected based on epidemiological data.

Slightly elevated rates of ischemic optic neuropathy (1.5%–1.7%) have been previously reported in clinical trials of complement inhibitors in GA.^17^^,^^49^^,^^50^ However, to the best of our knowledge, this is the first time that ischemic optic neuropathy has been reported at an incidence of more than 20%. In addition, a delayed onset of AEs more than 1 month after drug administration is uncommon. Accordingly, these safety signals should raise awareness for future clinical trials that ischemic optic neuropathy might be a drug-related AE with a delayed onset after intravitreal administration and long-term follow-up is necessary.

The pathological mechanisms behind the development of ischemic optic neuropathy after BI 754132 administration are not yet fully understood. Although there was no clear evidence of a dose–response relationship as events occurred at both high and low doses of BI 754132, 3 of the 4 participants who developed ischemic optic neuropathy were in a 6-mg dose group and one was in the 0.3-mg dose group. The presentation with a delayed onset after the injection makes it unlikely that the events were related to the injection procedure and potential postinjection IOP increases. Of note, the injected volumes of 50 μl (0.3-mg dose) and 100 μl (6-mg dose) are within the range of what is used in routine clinical practice. In addition, there was no evidence for longer-than-usual IOP increases associated with the study drug administration. A relevant observation was of thickening of the retina, predominantly in the outer retina, which was consistently observed on OCT in all cases. Such uniform changes in retinal thickness after intravitreal treatment are an uncommon finding. It may be hypothesized that the retinal thickening could have impaired optic nerve head perfusion and accounted for the increased risk for ischemic optic neuropathy observed in the current trial.

As demonstrated in the *in vitro* studies, TrkB agonism in SH-SY5Y cells increased the expression of genes regulating synaptic plasticity and induced dendritic growth. As such, the RNFL thickening may have been due to the apparent retinal axon branching and synaptogenesis that results from TrkB stimulation. Consistently, RNFL and retinal thickening were detected by OCT in the 26-week monkey intravitreal study. Similar retinal thickness findings were also reported in humans in another neuroprotective program using intravitreal delivery of ciliary neurotrophic factor, suggesting that this effect may be related to the neuroprotective action of neurotrophic factors.^51^ Other potential explanations include inflammation or an early neuroprotective reaction based on activation of retinal cells. However, the changes in retinal thickness observed in this phase I trial of BI 754132 were not associated with changes in retinal reflectivity or inflammation.

Interestingly, the increase in retinal thickness observed in cynomolgus monkeys after administration of BI 754132 was not associated with any changes to the optic nerve head. Furthermore, a ciliary neurotrophic factor–secreting intraocular NT-501 implant increased average RNFL thickness of study eyes by a mean of 10.16 μm (vs. 2.66 μm in the fellow eyes) but was well tolerated with no associated ischemic optic neuropathy reported.^52^ As such, the strength of the association between increased retinal thickness and the incidence of ischemic optic neuropathy requires further investigation. An alternative potential pathological mechanism may have been a direct effect of TrkB signaling (e.g., TrkB agonism or agonist-induced TrkB downregulation) on optic nerve head capillaries. Although there is currently no evidence supporting this in retinal disease, an independent trial evaluating TrkB agonism after subcutaneous administration of an antibody for cachexia in healthy participants was stopped early due to “sensory symptoms.”^53^ Although these data seem to suggest that TrkB agonism may have direct effects on nerve tissue that may lead to AEs, further research is needed to confirm this hypothesis.

Notably, ischemic optic neuropathy was not observed in cynomolgus monkey intravitreal injection studies. The discrepancy between the preclinical and clinical safety profiles of BI 754132 could be attributed to anatomic differences between animal models and the human eye and the lack of an animal model that fully represents GA or the aging human eye.^54^ Current animal models are limited to a single underlying pathological pathway, which may not fully represent GA pathophysiology or other pathophysiological features of the aging eye.^54^ Development of improved animal models of GA and the aging eye, combined with the use of detailed imaging techniques in preclinical studies, would improve the ability to determine if positive preclinical findings will translate to clinical settings. Future preclinical studies investigating novel TrkB agonists should consider performing RNFL and histopathological optic nerve analyses during follow-up to provide more insight into the pathophysiology and incidence of ischemic optic neuropathy events.

Although ischemic optic neuropathy generally presents with visual field defects or vision loss, animal models must experience severe vision loss or visual field defects for these to be recognized. In addition, small changes in retinal thickness or structure are usually difficult to detect with the conventional procedures used in toxicology assessments. Although the use of OCT in future preclinical studies may help to overcome the limitations associated with conventional procedures, more normative preclinical data are needed to identify mild pathological changes in retinal thickness and structure on OCT.

Although the results of the current study do not support further investigation of intravitreal BI 754132, they may help to inform the clinical development of TrkB agonists for other ophthalmic indications in the future. Regardless of outcomes from toxicology and preclinical studies, ischemic optic neuropathy should be considered a potential AE associated with new investigational agents with a related mode of action or method of administration in ophthalmology, calling to attention the importance of careful optic nerve head monitoring in early-phase trials. In addition, the current study highlights the importance of the proper design of first-in-human studies, avoiding exposing a high number of participants to an investigational agent unnecessarily, even if no safety signals are apparent from preclinical toxicology studies. This is of particular importance in studies investigating agents with potential new modes of action.

## Supplementary Data

Supplementary Material

Study group members
