# Supplementary material for: Tropomyosin-Related Kinase Receptor Type B Agonism in Geographic Atrophy—The Translational Challenges from Preclinical Data to a First-in-Human Trial
Source: Ophthalmol Sci. 2026 May 3;6(7):101216. doi: 10.1016/j.xops.2026.101216 (PMC13311265; doi:10.1016/j.xops.2026.101216)
Supplement: Figure S1 [file mmc1.pdf]

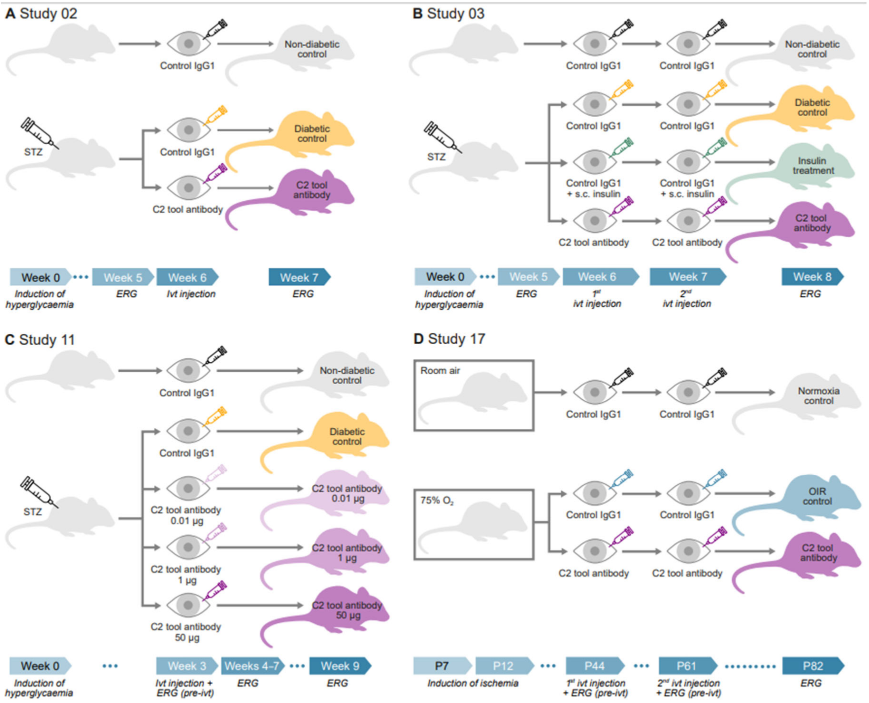

Figure S1. Summary of in vivo studies investigating the effects of TrkB agonism on retinal function restoration (A), preservation of retinal function (B), retinal neuroprotection in STZ-induced diabetic rats (C) and retinal function restoration in the OIR mouse mode (D). ERG= electroretinography; lgG1 = immunoglobulin G1; ivt = intravitreal; P = postnatal day; OIR = oxygen-induced retinopathy; s.c. = subcutaneous; STZ = streptozotocin; TrkB = tropomyosin--related kinase receptor type B.
