# Supplementary material for: Tropomyosin-Related Kinase Receptor Type B Agonism in Geographic Atrophy—The Translational Challenges from Preclinical Data to a First-in-Human Trial
Source: Ophthalmol Sci. 2026 May 3;6(7):101216. doi: 10.1016/j.xops.2026.101216 (PMC13311265; doi:10.1016/j.xops.2026.101216)
Supplement: Figure S2 [file mmc2.pdf]

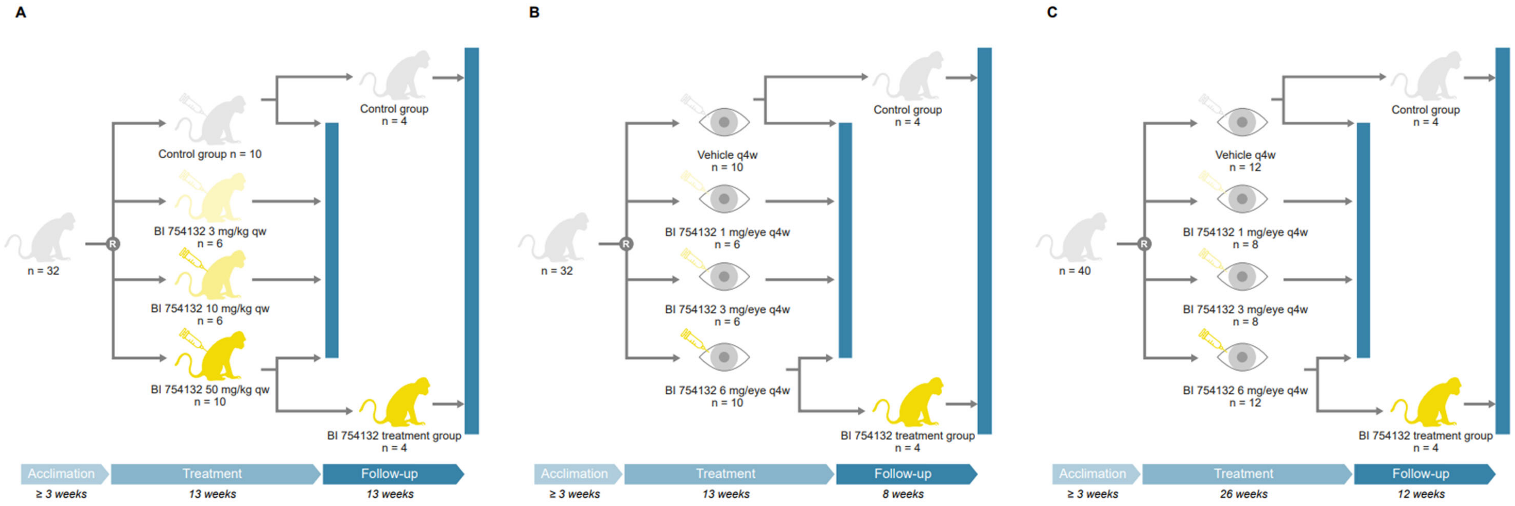

Figure S2. Summary of in vivo studies investigating lhe safely of intravenous (A) and intravitreal (B and C) B1 754132 in cynomolgus monkeys. Vertical blue boxes denote the point of euthanasia. q4w = once every 4 weeks; qw = once weekly; R = randomisation.
