# Supplementary material for: Tropomyosin-Related Kinase Receptor Type B Agonism in Geographic Atrophy—The Translational Challenges from Preclinical Data to a First-in-Human Trial
Source: Ophthalmol Sci. 2026 May 3;6(7):101216. doi: 10.1016/j.xops.2026.101216 (PMC13311265; doi:10.1016/j.xops.2026.101216)
Supplement: Figure S3 [file mmc3.pdf]

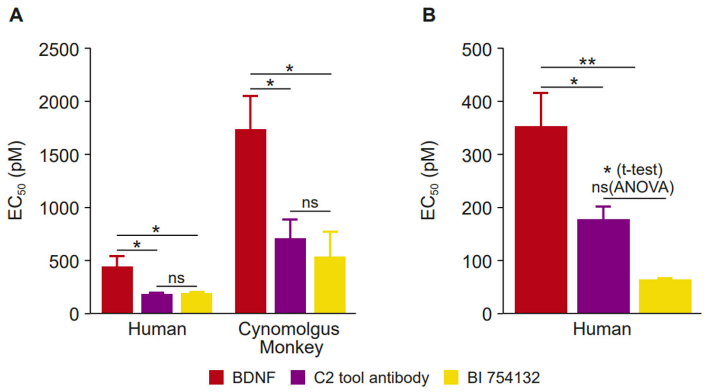

Figure S3. Mean potency of BDNF, C2 tool antibody and BI 754132 for TrkB in CHO cells overexpressing human and cynomolgus TrkB (A) and human neuronal SH-SY5Y cells (B). *$P < 0.05$; **$P < 0.01$ (one-way ANOVA with Tukey's multiple comparisons test; the data obtained for the C2 tool antibody and BI 754132 in human neuronal SH-SY5Y cells were additionally compared with the unpaired t-test as indicated). Error bars indicate SEM. ANOVA = analysis of variance; BDNF = brain-derived neurotrophic factor; CHO = Chinese hamster ovary; EC50 = half maximal effective concentration; ns = not significant; SEM = standard error of the mean; TrkB = tropomyosin-related kinase receptor type B.
