# Supplementary material for: Tropomyosin-Related Kinase Receptor Type B Agonism in Geographic Atrophy—The Translational Challenges from Preclinical Data to a First-in-Human Trial
Source: Ophthalmol Sci. 2026 May 3;6(7):101216. doi: 10.1016/j.xops.2026.101216 (PMC13311265; doi:10.1016/j.xops.2026.101216)
Supplement: Figure S4 [file mmc4.pdf]

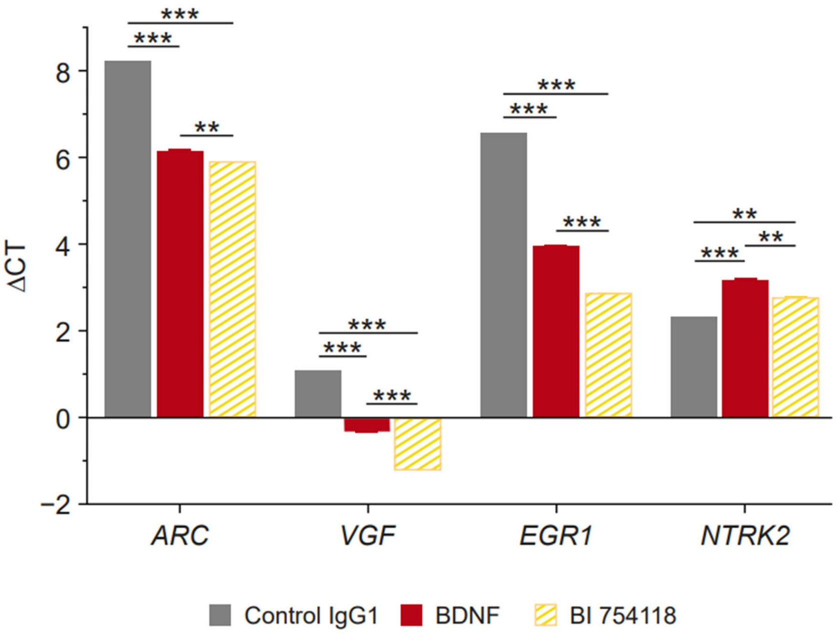

Figure S4. Effects of TrkB agonism on the regulation of mRNA levels in human neuronal SH-SYSY cells. ***P* < 0.01; ****P* < 0.001 (one-way ANOVA with Tukey's multiple comparisons test). Error bars indicate SEM. ANOVA = analysis of variance; BDNF = brain-derived neurotrophic factor; CT= cycle threshold; IgG1 = immunoglobulin G1; mRNA= messenger ribonucleic acid; SEM = standard error of the mean; TrkB = tropomyosin-related kinase receptor type B.
