# Supplementary material for: Tropomyosin-Related Kinase Receptor Type B Agonism in Geographic Atrophy—The Translational Challenges from Preclinical Data to a First-in-Human Trial
Source: Ophthalmol Sci. 2026 May 3;6(7):101216. doi: 10.1016/j.xops.2026.101216 (PMC13311265; doi:10.1016/j.xops.2026.101216)
Supplement: Figure S5 [file mmc5.pdf]

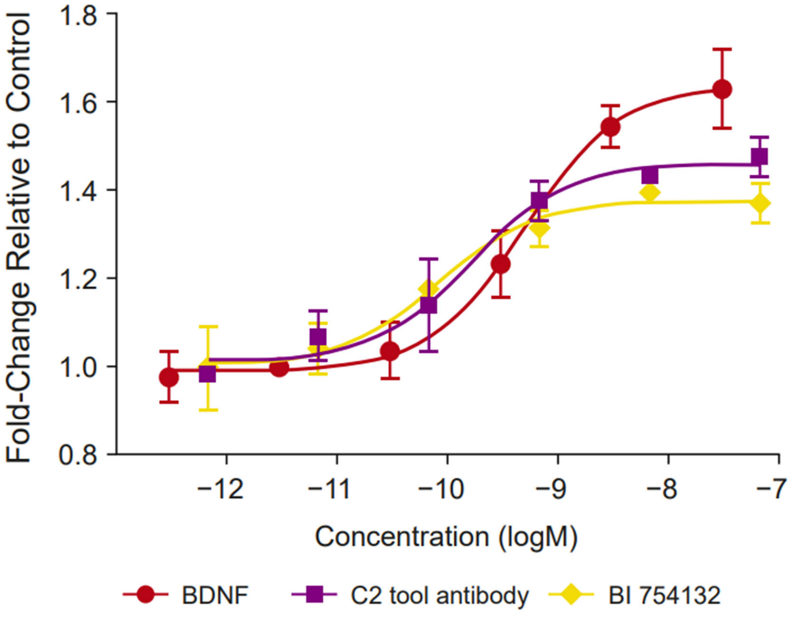

Figure S5. Formation of β3-tubulin-positive dendrites and synaptic connections induced by BDNF, C2 tool antibody and BI 754132 in human neuronal SH-SYSY cells. Error bars indicate SEM. BDNF = brain-derived neurotrophic factor; SEM = standard error of the mean.
