# Supplementary material for: Tropomyosin-Related Kinase Receptor Type B Agonism in Geographic Atrophy—The Translational Challenges from Preclinical Data to a First-in-Human Trial
Source: Ophthalmol Sci. 2026 May 3;6(7):101216. doi: 10.1016/j.xops.2026.101216 (PMC13311265; doi:10.1016/j.xops.2026.101216)
Supplement: Figure S6 [file mmc6.pdf]

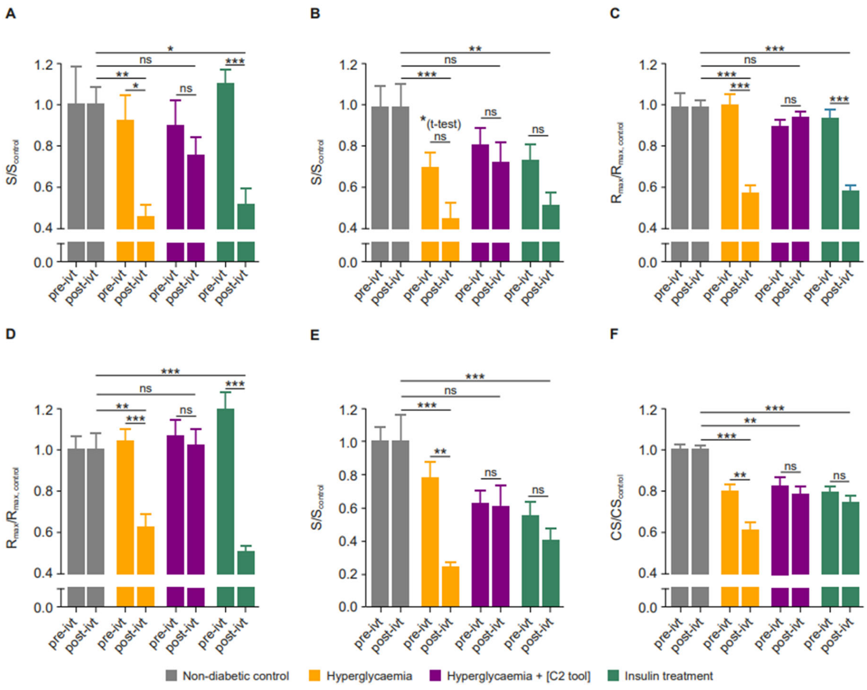

Figure S6. Mean value at each study point in rod-driven (A) and UV-cone-driven (B) b-wave light sensitivity, rod-driven (C) and UV-cone-driven (D) saturating b-wave response amplitude, UV-cone-driven photopic negative response (E) and outer retinal contrast sensitivity (F) in a preclinical study investigating the effects of TrkB agonism on retinal function presentation in streptozotocin-induced diabetic rats relative to non-diabetic control rats. *$P < 0.05$; **$P < 0.01$; ***$P < 0.001$ (one-way ANOVA with Tukey's multiple comparisons test; the data obtained for hyperglycaemlc control group at baseline and Week 8 were additionally compared with the paired t-test as indicated). Error bars indicate SEM. CS was determined by mixed rod- and cone-driven flicker ERG responses (at 12 Hz). ANOVA = analysis of variance; CS = temporal contrast sensitivity; CScontrol = temporal contrast sensitivity control; ERG = electroretinography; M = intravitreal; ns = not significant; R_{max} = saturating response amplitudes of rod-driven b-waves; $R_{max\ control}$ = R_{max} normalised to the mean saturating response amplitude of controls; S = light sensitivity; $S_{control}$ = light sensitivity normalised to the mean light sensitivities of controls; SME = standard error of the mean; TrkB = tropomyosin-related kinase receptor type B; UV = ultraviolet.
