# Supplementary material for: Tropomyosin-Related Kinase Receptor Type B Agonism in Geographic Atrophy—The Translational Challenges from Preclinical Data to a First-in-Human Trial
Source: Ophthalmol Sci. 2026 May 3;6(7):101216. doi: 10.1016/j.xops.2026.101216 (PMC13311265; doi:10.1016/j.xops.2026.101216)
Supplement: Figure S7 [file mmc7.pdf]

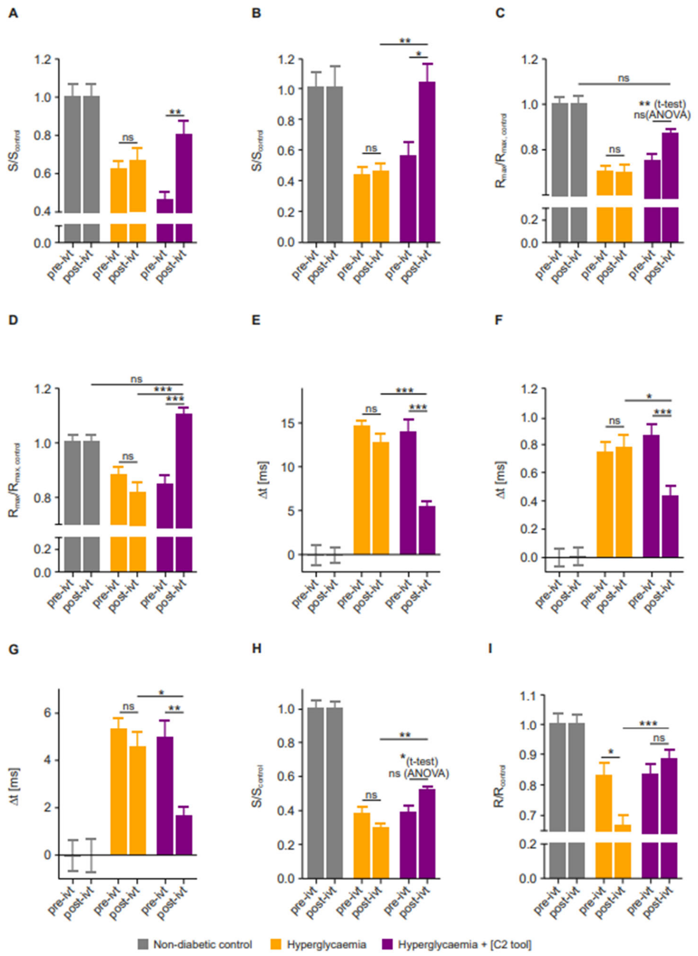

Figure S7. Mean value at each study point in rod-driven (A) and UV-cone-driven (B) b-wave light sensitivity, rod-driven (C) and M-cone-driven (D) saturating b-wave response amplitude, rod-driven (E), UV-cone-driven (F) and M-cone-driven (G) b-wave implicit times relative to the control group, the light sensitivity of the UV-cone-driven photopic negative response (H) and rod-driven a-wave responses (I) in a preclinical study investigating the effects of TrkB agonism on retinal function restoration in streptozotocin-induced diabetic rates relative to non-diabetic control rates. *$P < 0.05$; **$P < 0.01$; ***$P < 0.001$ (one-way ANOVA with Tukey's multiple comparisons test; the data obtained for the C2 tool antibody treatment group at baseline and Week 7 were additionally compared with the paired t-test, as indicated). Error bars indicate SEM. ANOVA = analysis of variance; ivt = intravitreal; ns = not significant; R_{max} = saturating response amplitudes of rod-driven b-waves; $R_{control}$ = normalised to the mean response of the non-diabetic control group; $R_{max\ control}$ = R_{max} normalised to the mean saturating response amplitude of controls; S = light sensitivity, $S_{control}$ = light sensitivity normalised to the mean light sensitivities of controls; SEM = standard error of the mean; TrkB = tropomyosin-related kinase receptor type B; UV = ultraviolet.
