# Supplementary material for: Tropomyosin-Related Kinase Receptor Type B Agonism in Geographic Atrophy—The Translational Challenges from Preclinical Data to a First-in-Human Trial
Source: Ophthalmol Sci. 2026 May 3;6(7):101216. doi: 10.1016/j.xops.2026.101216 (PMC13311265; doi:10.1016/j.xops.2026.101216)
Supplement: Figure S8 [file mmc8.pdf]

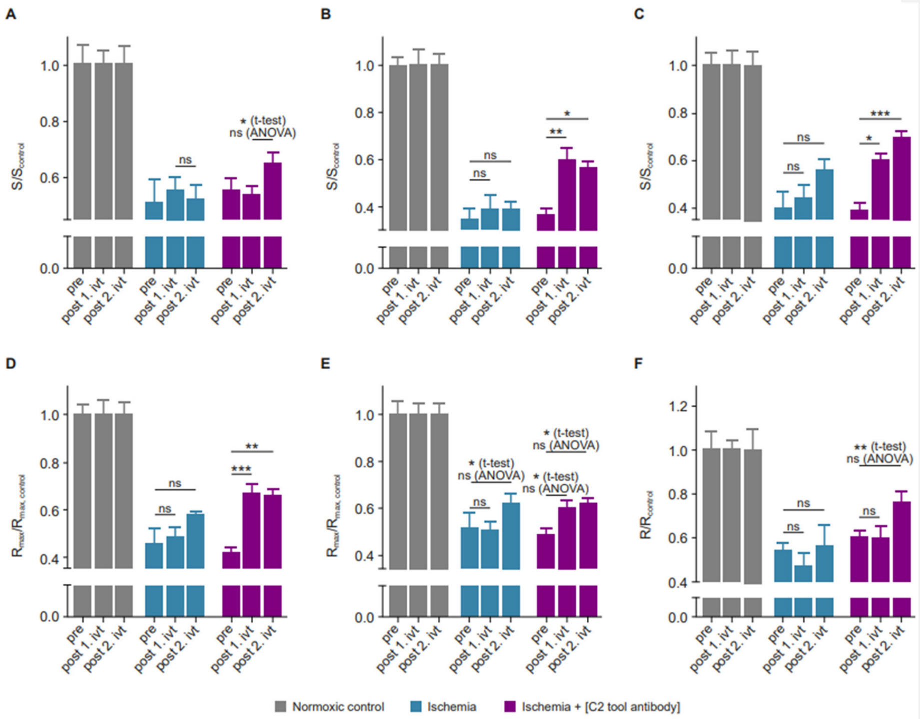

Figure S8. Mean value at each study point in rod-driven (A), UV-cone-driven (B) and M-cone-driven (C) b-wave light sensitivity, UV-cone-driven (D), M-cone-driven b-wave maximal oscillatory potentials (E) and cone-driven pattern ERG responses (F) in a preclinical study investigating the effects of TrkB agonism on retinal function restoration in OIR-induced ischemic mice relative to normoxic control mice. *$P < 0.05$; **$P < 0.01$; ***$P < 0.001$ (one-way ANOVA with Tukey's multiple comparisons test; paired t-test was also performed in some cases, as indicated). Error bars indicate SEM. ANOVA = analysis of variance; ERG = electroretinography; ivt = intravitreal; ns= not significant; OIR = oxygen-induced retinopathy; $R_{control}$ = normalised to the mean response of the non-diabetic control group; R_{max}= saturating response amplitudes of rod-driven b-waves; R_{max} = $R_{control}$ normalised to the mean saturating response amplitude of controls; S = light sensitivity; $S_{control}$ = light sensitivity normalised to the mean light sensitivities of controls; SEM = standard error of the mean; TrkB = tropomyosin-related kinase receptor type B; UV = ultraviolet.
