# Supplementary material for: Tropomyosin-Related Kinase Receptor Type B Agonism in Geographic Atrophy—The Translational Challenges from Preclinical Data to a First-in-Human Trial
Source: Ophthalmol Sci. 2026 May 3;6(7):101216. doi: 10.1016/j.xops.2026.101216 (PMC13311265; doi:10.1016/j.xops.2026.101216)
Supplement: Figure S9 [file mmc9.pdf]

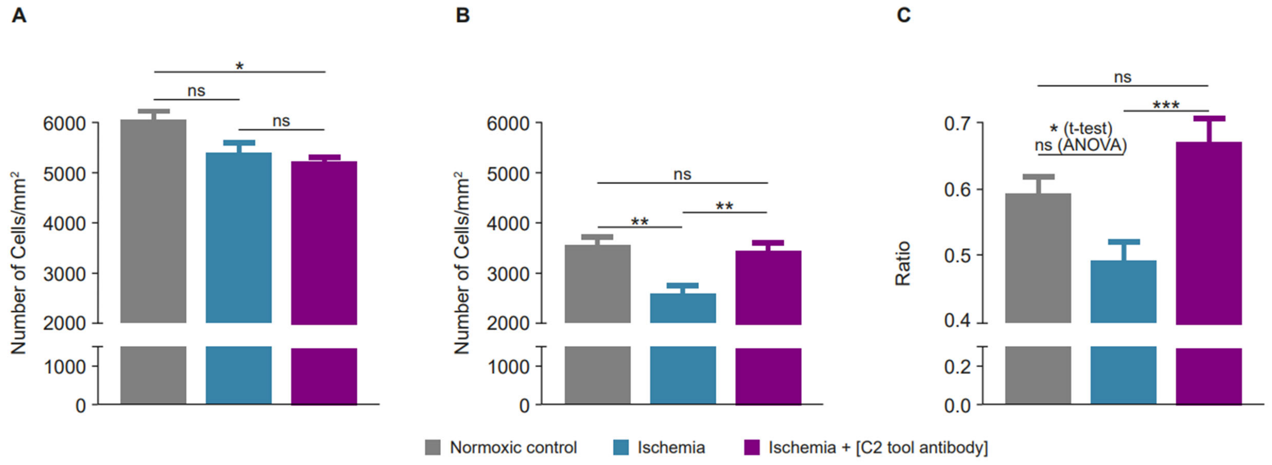

Figure S9. Number of DAPI-positive (A) and Brn3a-positive (B) cells and the ratio of Brn3a:DAPI positive cells (C) in retinal ganglion cell layer following intravitreal administration of C2 tool antibody or anti-TNP control antibody in OIR-induced ischemic mice relative to normoxic controls. *$P < 0.05$; **$P < 0.01$; ***$P < 0.001$ (one-way ANOVA with Tukey's multiple comparisons test; paired t-test was also performed in some cases, as indicated). Error bars indicate SEM. ANOVA = analysis of variance; DAPI = 4',6-diamidino-2-phenylindole; ns = not significant; OIR = oxygen-induced retinopathy; SEM = standard error of the mean; TNP = trinitrophenol.
