# Supplementary material for: Tropomyosin-Related Kinase Receptor Type B Agonism in Geographic Atrophy—The Translational Challenges from Preclinical Data to a First-in-Human Trial
Source: Ophthalmol Sci. 2026 May 3;6(7):101216. doi: 10.1016/j.xops.2026.101216 (PMC13311265; doi:10.1016/j.xops.2026.101216)
Supplement: Figure S10 [file mmc10.pdf]

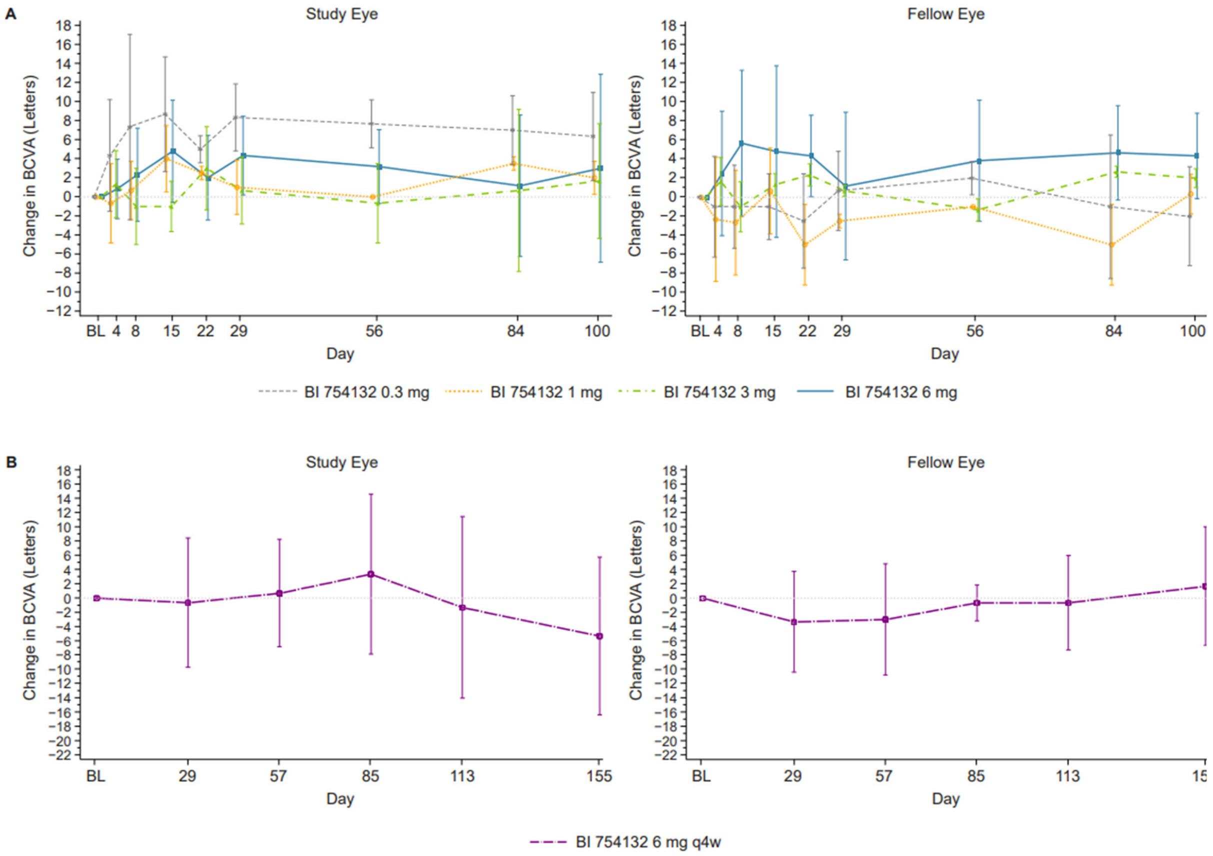

Figure S10. Mean change from baseline in BCVA of the study (left) and fellow eyes (right) over time in the SRD (A) and MD (B) parts of the Phase I trial (TS). Error bars show SD. BL was defined as last measurement before the first administration of BI 754132. BCVA = best corrected visual acuity; BL = baseline; MD = multiple dose; q4w = administration 4 times weekly; SD = standard deviation; SRD = single rising dose; TS = treated set.
