# Supplementary material for: Tropomyosin-Related Kinase Receptor Type B Agonism in Geographic Atrophy—The Translational Challenges from Preclinical Data to a First-in-Human Trial
Source: Ophthalmol Sci. 2026 May 3;6(7):101216. doi: 10.1016/j.xops.2026.101216 (PMC13311265; doi:10.1016/j.xops.2026.101216)
Supplement: Figure S11 [file mmc11.pdf]

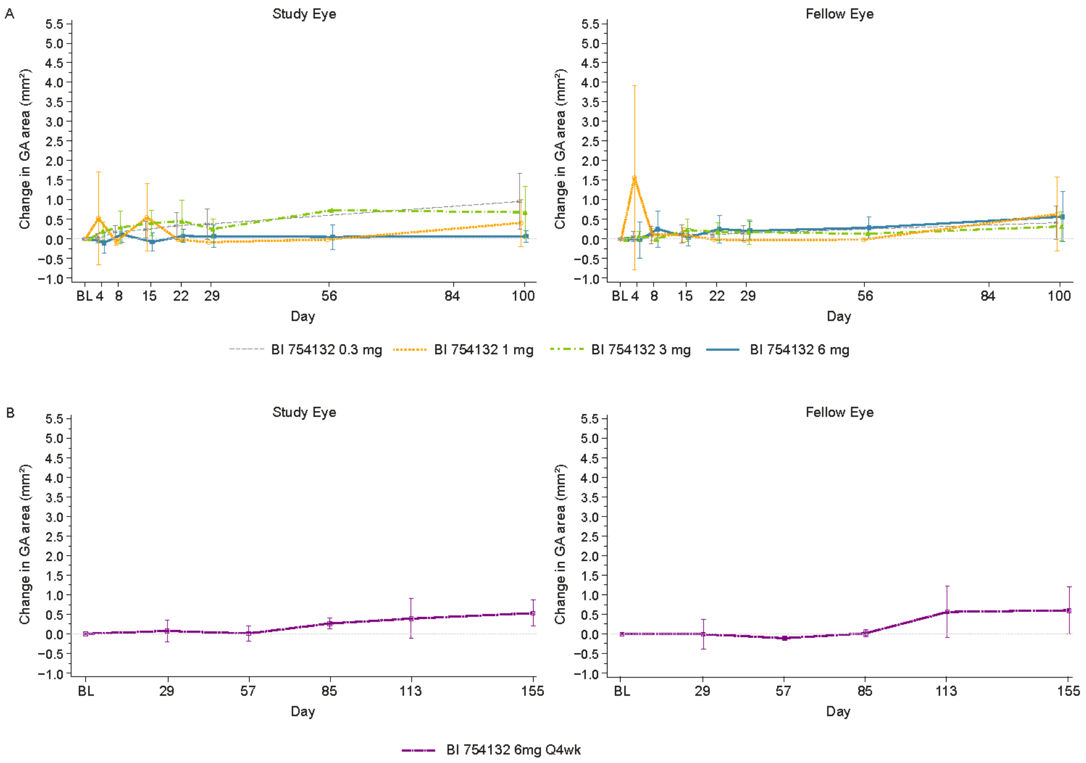

Fig S11. Mean change from baseline in GA area in the study (left) and fellow eyes (right) over time in the SRD (A) and MD (B) parts of the Phase I trial (TS). Error bars show SD. BL was defined as treatment Visit 2 (Day 1). If no BL value was available at Visit 2, BL was defined as the last measurement taken at screening (Visit 1; Day -3). GA = geographic atrophy; MD = multiple dose; Q4wk = administration 4 times weekly; SD = standard deviation; SRD = single rising dose; TS = treated set.
