# Supplementary material for: Tropomyosin-Related Kinase Receptor Type B Agonism in Geographic Atrophy—The Translational Challenges from Preclinical Data to a First-in-Human Trial
Source: Ophthalmol Sci. 2026 May 3;6(7):101216. doi: 10.1016/j.xops.2026.101216 (PMC13311265; doi:10.1016/j.xops.2026.101216)
Supplement: Figure S12 [file mmc12.pdf]

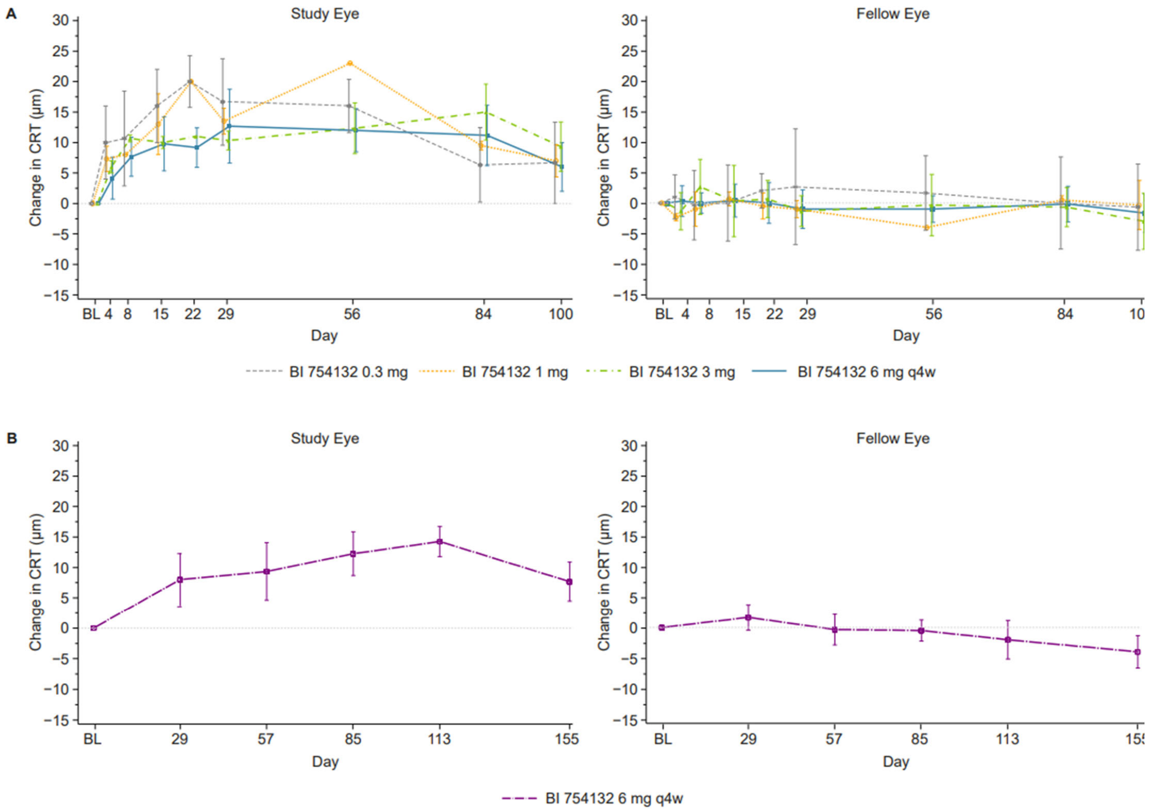

Figure S12. Mean change from baseline in CRT of the study (left) and fellow eyes (right) over time in the SRD (A) and MD (B) parts of the Phase I trial (TS). Error bars show SD. BL was defined as treatment Visit 2 (Day 1). If no BL value was available at Visit 2, BL was defined as the last measurement taken at screening (Visit 1; Day -3). BL = baseline; CRT, central retinal thickness; MD = multiple dose; q4w= administration 4 times weekly; SD = standard deviation; SRD = single rising dose; TS = treated set.
