# Supplementary material for: Tropomyosin-Related Kinase Receptor Type B Agonism in Geographic Atrophy—The Translational Challenges from Preclinical Data to a First-in-Human Trial
Source: Ophthalmol Sci. 2026 May 3;6(7):101216. doi: 10.1016/j.xops.2026.101216 (PMC13311265; doi:10.1016/j.xops.2026.101216)
Supplement: Figure S14 [file mmc14.pdf]

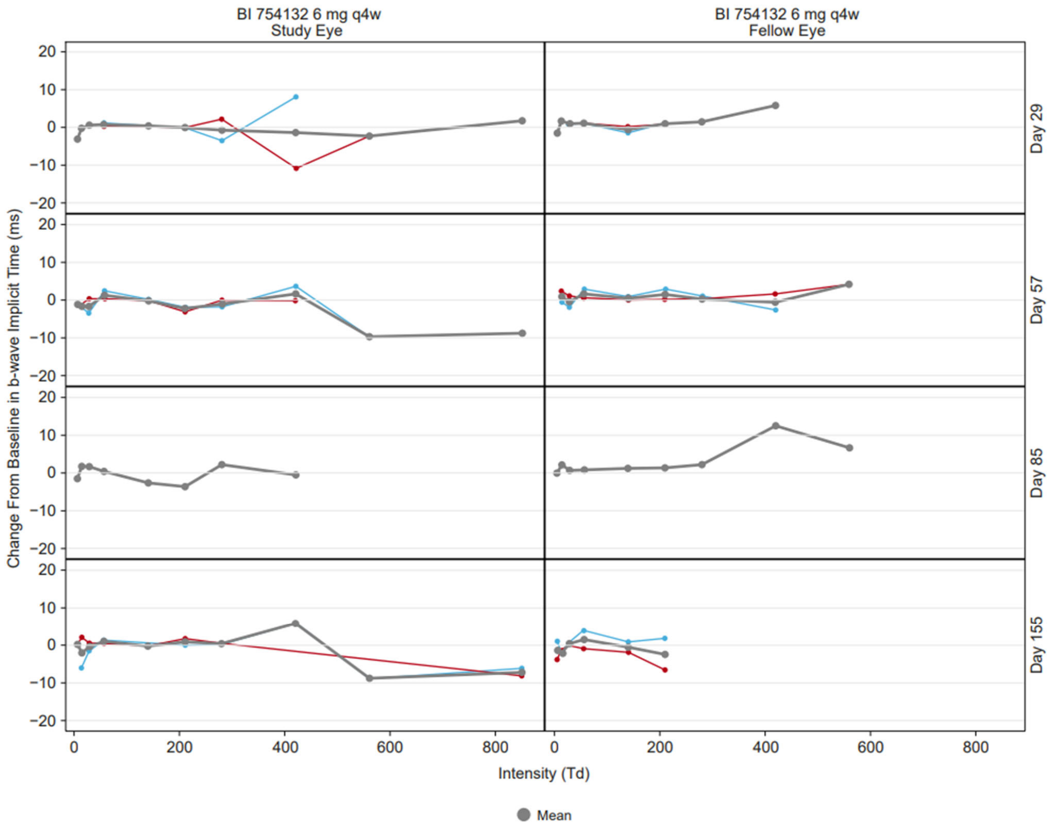

Figure S14. Changes from baseline in b-wave implicit time in response to different flash intensity in the MD part of the Phase I trial (TS). Each line represents one subject. MD = multiple dose; q4w= administration 4 times weekly; TS = treated set.
