# Supplementary material for: Tropomyosin-Related Kinase Receptor Type B Agonism in Geographic Atrophy—The Translational Challenges from Preclinical Data to a First-in-Human Trial
Source: Ophthalmol Sci. 2026 May 3;6(7):101216. doi: 10.1016/j.xops.2026.101216 (PMC13311265; doi:10.1016/j.xops.2026.101216)
Supplement: Table S1 [file mmc15.pdf]

Table S1. *In Vitro* Affinity of the C2 Tool Antibody and BI 754132 for Human TrkB

**Parameter**	**C2 Tool Antibody**	**BI 754132**
Kd, nM	8.40	1.44
Kon, M-1s-1	39,400	220,000
Koff, s^{-1}	0.000332	0.000317

Kd = dissociation constant; Koff = dissociation rate constant; Kon = association rate constant; TrkB = tropomyosin-related kinase receptor type B.
