# Supplementary material for: Tropomyosin-Related Kinase Receptor Type B Agonism in Geographic Atrophy—The Translational Challenges from Preclinical Data to a First-in-Human Trial
Source: Ophthalmol Sci. 2026 May 3;6(7):101216. doi: 10.1016/j.xops.2026.101216 (PMC13311265; doi:10.1016/j.xops.2026.101216)
Supplement: Table S2 [file mmc16.pdf]

Table S2. Effects of BDNF, the C2 Tool Antibody and BI 754132 on TrkB Phosphorylation and Intracellular Signalling in CHO Cells Overexpressing Human TrkB

	**BDNF**		**C2 Tool Antibody**		**BI 754132**	
**Parameter, Mean (SEM)**	**EC50 (pM)**	**Emax (% of BDNF)**	**EC50 (pM)**	**Emax (% of BDNF)**	**EC50 (pM)**	**Emax (% of BDNF)**
TrkB phosphorylation	420 (110)[a]	100 (0)[a]	162 (24)[b]	43 (2)[b]	172 (20)[b]	51 (2)[b]
AKT1/2/3 phosphorylation	220 (67)[c]	100 (0)[c]	47 (21)[d]	101 (11)[d]	30 (2)[d]	72 (3)[d]
ERK1/2 phosphorylation	92 (28)[e]	100 (0)[e]	34 (10)[a]	88 (5)[a]	53 (26)[f]	76 (11)[f]
IP1 accumulation	461 (122)[c]	100 (0)[c]	134 (21)[f]	57 (7)[f]	115 (20)[f]	59 (7)[f]

[a]n=7; [b]n=10; [c]n=3; [d]n=2; [e]n=6; [f]n=4. Emax is given as % of the maximal effect induced by saturating doses of BDNF.
AKT1/2/3 = serine/threonine kinase AKT isoforms 1/2/3; BDNF = brain-derived neurotrophic factor; CHO = Chinese hamster ovary; EC50 = half maximal effective concentration; Emax = maximal inducible effect; ERK1/2 = extracellular signal-regulated kinase 1/2; IP1 = inositol-1-phosphate; SEM = standard error of the mean; TrkB = tropomyosin-related kinase receptor type B.
