# Supplementary material for: Tropomyosin-Related Kinase Receptor Type B Agonism in Geographic Atrophy—The Translational Challenges from Preclinical Data to a First-in-Human Trial
Source: Ophthalmol Sci. 2026 May 3;6(7):101216. doi: 10.1016/j.xops.2026.101216 (PMC13311265; doi:10.1016/j.xops.2026.101216)
Supplement: Table S3 [file mmc17.pdf]

Table S3. Effects of BDNF, the C2 Tool Antibody, BI 754132, and BI 754118 on TrkB Phosphorylation and Intracellular Signalling in Human Neuronal SH-SY5Y Cells

	**BDNF**		**C2 Tool Antibody**		**BI 754132**		**BI 754118**	
**Parameter, Mean (SEM)**	**EC50 (pM)**	**Emax (% of BDNF)**	**EC50 (pM)**	**Emax (% of BDNF)**	**EC50 (pM)**	**Emax (% of BDNF)**	**EC50 (pM)**	**Emax (% of BDNF)**
TrkB phosphorylation	349 (65)[a]	99 (6)[a]	171 (29)[b]	78 (7)[b]	58 (7)[c]	38 (6)[c]	140 (37)[d]	83 (9)[d]
AKT1/2/3 phosphorylation	253 (53)[b]	100 (1)[b]	227 (46)[e]	110 (10)[e]	32 (3)[c]	75 (2)[c]	61 (23)[f]	104 (9)[f]
ERK1/2 phosphorylation	247 (63)[g]	104 (4)[g]	131 (21)[d]	106 (7)[d]	27 (4)[c]	113 (2)[c]	163 (58)[h]	102 (8)[h]

[a]n=16; [b]n=14; [c]n=6; [d]n=11; [e]n=8; [f]n=7; [g]n=15; [h]n=10. Emax is given as % of the maximal effect induced by saturating doses of BDNF.
AKT1/2/3 = AKT serine/threonine kinase 1/2/3; BDNF = brain-derived neurotrophic factor; EC50 = half-maximal effective concentration; Emax = maximal inducible effect; ERK1/2 = extracellular signal-regulated kinase 1/2; SEM = standard error of the mean; TrkB = tropomyosin-related kinase receptor type B.
