# Supplementary material for: Tropomyosin-Related Kinase Receptor Type B Agonism in Geographic Atrophy—The Translational Challenges from Preclinical Data to a First-in-Human Trial
Source: Ophthalmol Sci. 2026 May 3;6(7):101216. doi: 10.1016/j.xops.2026.101216 (PMC13311265; doi:10.1016/j.xops.2026.101216)
Supplement: Table S4 [file mmc18.pdf]

Table S4. Toxicokinetic Profile of Intravenous BI 754132 in Cynomolgus Monkeys

Study	Treatment Group	PK Parameter, Mean (SD)					
		C_{max}, µg/mL		AUC, µg•h/mL[a]		t_{max}, h[b]	
		Baseline	Week 13	Baseline	Week 8	Baseline	Week 8
Intravenous injection study	BI 754132 3 mg/kg qw (n=6)	78.8 (12.6)	142 (24.3)	5,450 (764)	13,200 (2,130)	0.083 (0.083–0.083)	0.083 (0.083–8)
	BI 754132 10 mg/kg qw (n=6)	307 (110)	523 (90.5)	20,300 (3,610)	56,500 (12,700)	0.083 (0.083–8)	0.083 (0.083–24)
	BI 754132 50 mg/kg qw (n=10)	1,260 (271)	2,270 (325)	99,200 (18,100)	252,000 (47,600)	0.083 (0.083–8)	0.083 (0.083–8)
13-week intravitreal injection study	BI 754132 1 mg/eye q4w (n=6)	1.22 (0.251)	0.543 (0.364)	161 (27.8)	73.9 (64.2)[c]	24 (24–72)	72 (72–72)[c]
	BI 754132 3 mg/eye q4w (n=6)	5.21 (1.12)	4.49 (1.20)[d]	829 (222)	744 (202)[d]	48 (24–72)	72 (72–72)[d]
	BI 754132 6 mg/eye q4w (n=10)	12.9 (3.87)	11 (2.65)	2,480 (801)	2,060 (527)	72 (24–72)	72 (72–72)

[a]AUC of BI 754132 was calculated over the time interval from 0 to 168 hours post-dose in the intravenous injection study and 0–672 hours post-dose in the intravitreal injection study; [b]Presented as median (range); [c]n=4; [d]n=5. AUC = area under the concentration–time curve; C_{max} = maximum serum concentration of BI 754132 after a single intravitreal dose; PK = pharmacokinetics; q4w = once every 4 weeks; qw = once weekly; SD = standard deviation; t_{max} = time from dosing to maximum serum concentration of BI 754132.
