# Supplementary material for: Tropomyosin-Related Kinase Receptor Type B Agonism in Geographic Atrophy—The Translational Challenges from Preclinical Data to a First-in-Human Trial
Source: Ophthalmol Sci. 2026 May 3;6(7):101216. doi: 10.1016/j.xops.2026.101216 (PMC13311265; doi:10.1016/j.xops.2026.101216)
Supplement: Table S5 [file mmc19.pdf]

Table S5. Selection Criteria for the Clinical Trial of BI 754132

	**Inclusion Criteria**
Both trial parts	Male or female participants aged ≥50 years with GA secondary to AMD in ≥1 eye with signed and dated written informed consent<br>Better BCVA score in the study eye than in the fellow eye
SRD part	GA lesion in the study eye ≥1.9 mm^2 (approximately ≥0.75 disc area in size)<br>BCVA in the study eye between 20/100 and 20/400 (19–53 letters on the ETDRS chart) measured according to the ETDRS protocol
MD part	Total GA lesion size in the study eye of ≥7.5 mm^2 (approximately ≥3 disc area in size)<br>BCVA in the study eye of 20/100 (≤53 letters on the ETDRS chart), measured according to the ETDRS protocol
	**Exclusion Criteria**
Female participants	Female participants of childbearing potential
Male participants	Fertile male participants unwilling or unable to use highly effective methods of birth control, with a failure rate ≤1% per year when used consistently and correctly
MD part	Previous participation in the SRD part of the current trial<br>Unstable and/or untreated cardiovascular or metabolic disease (e.g. hypertension, diabetes mellitus or hypercholesterolaemia) within ≥3 months of screening<br>Any evidence of current or past giant cell arteritis
Diagnosis	GA due to causes other than AMD in the study or fellow eye<br>History of choroidal neovascularisation in the study and in the fellow eye<br>Any other eye disease in the study eye that could compromise:<br>• BCVA or result in visual field loss<br>• Uncontrolled glaucoma IOP (defined as IOP >24)<br>• Clinically significant diabetic maculopathy<br>• History of ischemic optic neuropathy or retinal vascular occlusion<br>• Symptomatic vitreomacular traction<br>• Genetic disorders, such as retinitis pigmentosa<br>• History of high myopia of >8 dioptres in the study eye<br>• Anterior segment and vitreous abnormalities in the study eye that would preclude adequate observation with SD-OCT

	Aphakia or total absence of the posterior capsule
	Malignancy for which the participant has undergone resection, radiation or chemotherapy within past 5 years (except treated basal cell carcinoma or fully cured squamous cell carcinoma)
	Significant disease or medical conditions at screening that may, in the opinion of the investigator, put the participant at risk, influence the results of the study or cause concern regarding the participant’s ability to participate in the study
	Active intraocular inflammation in the study eye
	Active infectious conjunctivitis in the study or fellow eye
Concomitant therapy	Treatment for GA secondary to AMD in the study eye within ≤6 months of screening (except vitamin and mineral supplements)
	YAG laser capsulotomy in the study eye ≤3 months prior to enrolment
	Current or planned use of medications known to be toxic to the retina, lens or optic nerve (e.g. deferoxamine, chloroquine or hydroxychloroquine, chlorpromazine, phenothiazines, tamoxifen, nicotinic acid and ethambutol)
Other	Individuals under administrative/legal supervision or institutionalisation due to a regulatory or juridical order
	Previous participation in other clinical trials evaluating a treatment for GA
	Prior intraocular surgery in the study eye other than uneventful lens replacement for cataract within ≤3 months of screening
	Known hypersensitivity to any of the ingredients used in the IMP formulation, or any of the medications used

AMD = age-related macular degeneration; BCVA = best corrected visual acuity; ETDRS = Early Treatment Diabetic Retinopathy Study; GA = geographic atrophy; IMP = investigational medical product; IOP = intraocular pressure; MD = multiple dose; SD-OCT = spectral-domain optical coherence tomography; SRD = single-rising dose; YAG = yttrium aluminium garnet.
