# Supplementary material for: Tropomyosin-Related Kinase Receptor Type B Agonism in Geographic Atrophy—The Translational Challenges from Preclinical Data to a First-in-Human Trial
Source: Ophthalmol Sci. 2026 May 3;6(7):101216. doi: 10.1016/j.xops.2026.101216 (PMC13311265; doi:10.1016/j.xops.2026.101216)
Supplement: Supplementary Material [file mmc20.pdf]

Supplemental Material

Tropomyosin-Related Kinase Receptor Type B Agonism in Geographic Atrophy – The Translational Challenges From Preclinical Data to a First-in-Human Trial

David Brown, Oliver Zeitz, Clare Bailey, Sunir Garg, Karl Csaky, James Talks, Sobha Sivaprasad, Peter M. Benz, Rolf Herrmann, Remko A. Bakker, Sebastian Bandholtz, Ankit Mittal, Qihong Huang, Serge Kosobokovs, Gudrun Simons, Andrea Giani, Jochen Huber, Martin Gliem, on behalf of the 1418.01 study group

Contents

Preclinical Methodology 2

In Vitro Studies 2

In Vivo Studies 5

Statistical Analysis of Preclinical Data 10

Clinical Methodology 11

Randomisation and Masking 11

Preclinical Methodology

In Vitro Studies

Characterisation of the affinity and selectivity of BI 754132, the C2 tool antibody and brain-derived neurotrophic factor (BDNF) for tropomyosin-related kinase receptor type B (TrkB) in Chinese hamster ovary (CHO) cells

CHO cells, suspended in Dulbecco's Modified Eagle's Medium (DMEM; 4.5 g glucose/L; Lonza Group, Basel, Switzerland) supplemented with 10% foetal calf serum (FCS; Biological Industries, Kibbutz Beit Haemek, Israel), 1 × GlutaMAX™ (Thermo Fisher Scientific, Waltham, MA, USA), 1 × non-essential amino acid solution (Lonza Group), 20 mM HEPES (Lonza Group), 5 µg/mL blasticidin S (Thermo Fisher Scientific) and 200 µg/mL Zeocin™ (Thermo Fisher Scientific), were cultured in a humidified incubator at 37°C and 5% CO_2.

After 24 hours, the medium was exchanged with 80 µL of DMEM with 0.2% bovine serum albumin (BSA; Sigma-Aldrich, St Louis, MO, USA) and cells were incubated at room temperature (RT) for 15 minutes. Subsequently, 20 µL of BI 754132, the C2 tool antibody or BDNF (R&D Systems, Minneapolis, MN, USA; Lot: NG6815051) were added. After 45 minutes of incubation at RT, the medium was removed and cells were incubated in 30 µL of ice-cold lysis buffer for 20 minutes.

To determine the extent of neurotrophic tyrosine kinase receptor (Trk) phosphorylation in the lysed cells, 2.5 µL of the cell lysate was initially incubated (45 minutes; RT) in the dark with 5 µL of phospho-Trk acceptor beads (PerkinElmer, Waltham, MA, USA), diluted in AlphaLISA™ immunoassay (PerkinElmer) to 10 µg/mL, and subsequently incubated with 2.5 µL of Trk-pan biotin antibodies (PerkinElmer), diluted in AlphaLISA immunoassay to 1 nM. Following this incubation, 2.5 µL of streptavidin-donor beads (PerkinElmer), diluted in AlphaLISA immunoassay to 20 µg/mL, were added and the lysate was stored for 30 minutes at RT in the dark. Subsequently, the extent of

phosphorylation was measured with an EnVision™ plate reader (PerkinElmer) using the AlphaScreen® protocol (filter 570 nm #244 and mirror #444).

Further tests included elucidation of the extent of the serine/threonine kinases AKT 1, AKT 2 and AKT 3 (AKT1/2/3) and extracellular signal-regulated kinase 1/2 (ERK1/2) phosphorylation in the cell lysate (8 µL) using the AlphaScreen SureFire p-AKT1/2/3 and p-ERK1/2 assays (PerkinElmer), respectively, performed according to the manufacturer's instructions. In addition, the accumulation of inositol-1-phosphate was evaluated via the IP-One HTRF® assay (Cisbio Bioassays, Codolet, France).

Characterisation of the effects of TrkB agonism on signalling in human neuronal SH-SY5Y cells

SH-SY5Y cells (ATCC, Manassas, VA, USA) suspended in DMEM/F-12 medium (Lonza Group) supplemented with 15% FCS (Biological Industries, Kibbutz Beit Haemek, Israel) were cultured in a humidified incubator at 37°C, 5% CO_2 for 8 hours. Subsequently, the cells were mixed with 6 µM retinoic acid (Sigma-Aldrich), so that the final retinoic acid concentration was 3 µM. After 48 and 86 hours, cells were re-suspended in fresh medium, and retinoic acid stimulation was re-performed. After 7 days of differentiation, SH-SY5Y cells developed a dopaminergic-like phenotype and were ready to use.

For the phosphorylation assays, following SH-SY5Y cell differentiation, the medium was exchanged with 80 µL of pre-warmed DMEM/F-12 supplemented with 0.2% BSA, and cells were incubated at RT for 15 minutes. Subsequently, 20 µL of peptide/antibody was added and cells were incubated for a further 45 minutes at RT. To stimulate cell lysis, the medium was removed and cells were incubated in 30 µL of ice-cold lysis buffer for 20 minutes. The extent of TrkB (Y706/Y707), AKT1/2/3 and ERK1/2 phosphorylation in the cell lysate was subsequently determined as previously described for CHO cells.

The differentiated SH-SY5Y cells were also used to measure changes in mRNA levels of genes regulating synaptic function (*ACTB*, *NTRK2*, *ARC*, *VGF* and *EGR1*) via real-time quantitative PCR. Differentiated SH-SY5Y cells, prepared as previously described, were stimulated by incubation (humidified incubator; 37°C, 5% CO_2) in serum-free DMEM/F-12 (0.2% BSA) with the control anti-

trinitrophenol (TNP) antibody (10 nM), BDNF (10 nM) or BI 754118 (10 nM) for 6 hours. mRNA isolation was performed using the RNeasy® Mini Kit (QIAGEN, Hilden, Germany) and the transcription to complementary DNA using the QuantiTect Reverse Transcription kit (QIAGEN), according to the manufacturer's instructions. The complementary DNA was subsequently used for real-time PCR assays (TaqMan™; Thermo Fisher Scientific), performed using a fast real-time PCR system (7900HT; Applied Biosystems, Waltham, MA, USA).

For quantification of dendrite and synapse formation, dendrites and synaptic connections were labelled by β3-tubulin staining. Human neuronal SH-SY5Y cells, prepared as previously described, were stimulated by incubation (humidified incubator; 37°C, 5% CO_2) in serum-free DMEM/F-12 supplemented with 15% FCS, with different concentrations of BDNF, the C2 tool antibody or BI 754132 for 3 days. Following stimulation, the medium was removed and cells were fixed by applying paraformaldehyde (4% in phosphate-buffered saline [PBS; Thermo Fisher Scientific]) for 15 minutes at RT. After each subsequent step, the fixed cells were washed twice with PBS. The fixed cells were permeabilised (5 minutes, RT, 0.1% Triton™ X-100 [Cell Signaling Technology, MA, USA]) and incubated (60 minutes) with BSA (5% in PBS) to block non-specific binding sites. Subsequently, the cells were incubated with anti-β3-tubulin (Sigma-Aldrich) and diluted 1:200 in 0.5% BSA overnight at 4°C. Following incubation, cells were stained (HCS NuclearMask™ Blue stain [Invitrogen, Waltham, MA, USA]) and incubated with goat anti-mouse immunoglobulin G (IgG) antibodies labelled with Alexa Fluor 488 (1:400 in 0.5% BSA/PBS) for 30 minutes at RT. The cells were then analysed with a fully automated microscope (BD Pathway 855; Olympus 20 × objective). Quantification was performed with BS AttoVision (BD Biosciences, Franklin Lakes, NJ, USA). Areas of nuclei and β3-tubulin were calculated as the number of pixels; the β3-tubulin area was normalised to the nuclei area.

In Vivo Studies

Ethical approval

All preclinical experimental protocols were reviewed by a federal ethics committee and were reviewed and approved by the governmental body responsible for animal welfare in the state of Baden-Württemberg (Regierungspräsidium Tübingen, Germany). The studies adhered to the Guide for the Care and Use of Laboratory Animals and the Animal Research: Reporting of In Vivo Experiments (ARRIVE) guidelines, to the Association for Research in Vision and Ophthalmology Statement for the Use of Animals in Ophthalmic and Vision Research, and were carried out in accordance with the Guide for the Care and Use of Laboratory Animals, as adopted and promulgated by National Institutes of Health.

Effects of TrkB agonism (C2 tool antibody) on retinal function in streptozotocin-induced diabetic rats

Hyperglycaemia was induced by intraperitoneal streptozotocin injection (65 mg/kg bodyweight; Sigma-Aldrich) in 6–8-week-old male Brown Norway rats (n=24 rats; Charles River Laboratories, Erkrath, Germany); 12 rats did not receive the streptozotocin injection, to act as non-diabetic controls (Group 1). The rats were housed in pairs in individually ventilated cages maintained at controlled temperature and humidity conditions, with a 12-hour light/dark cycle and *ad libitum* access to food and water for 1 week before the start of the study and throughout the study period. Unresponsive animals (blood glucose <20 mM following streptozotocin injection) were excluded from the preclinical studies.

The effect of the C2 tool antibody on retinal function restoration was investigated in 24 streptozotocin-induced diabetic rats and 12 non-diabetic control rats. There were three treatment groups: the C2 tool antibody treatment group (n=12 rats), diabetic control group (n=12 rats) and non-diabetic control group (n=12 rats). At the start of Week 6, rats in the C2 tool antibody treatment group received a single intravitreal injection of the C2 tool antibody (4.6 µL; 10.9 mg/mL), and rats in

the non-diabetic and diabetic control groups were administered a single intravitreal dose of an IgG1 control antibody (anti-TNP; 4.5 µL; 11.2 mg/mL). To assess the extent of retinal function restoration, changes from pre-intravitreal to 1 week post-intravitreal were investigated in rod- and UV-cone–driven b-wave light sensitivity; rod- and M-cone-driven saturating b-wave response amplitude; rod-, UV-cone- and M-cone-driven b-wave implicit times; UV-cone-driven photopic negative response; and rod-driven a-wave responses.

Subsequently, the effect of the C2 tool antibody on the preservation of retinal function of streptozotocin-induced diabetic rats was investigated in comparison with insulin treatment. This experiment was conducted in 35 streptozotocin-induced hyperglycaemic rats and 10 non-diabetic rats. There were four treatment groups: C2 tool antibody treatment group (n=12 rats), diabetic control group (n=11 rats), insulin treatment group (n=12 rats) and non-diabetic control group (n=10 rats). At Days 35 and 46 post-hyperglycaemia induction, rats in the diabetic control, non-diabetic control and insulin treatment groups received 5 µL of intravitreal anti-TNP (2.73 mg/mL), and rats in the C2 tool antibody treatment group received 4.5 µL of intravitreal C2 tool antibody (11.35 mg/mL). Rats randomised to the insulin treatment group were also administered subcutaneous insulin pellets at the start of Week 6. To assess the preservation of retinal function, changes from baseline to Week 8 were investigated in rod- and UV-cone-driven b-wave light sensitivities, rod- and UV-cone-driven saturating b-wave amplitude, UV-cone-driven photopic negative response, and contrast sensitivities.

Finally, the duration of retinal neuroprotection following C2 tool antibody administration (0.01, 1 or 50 µg/eye) was investigated in 48 streptozotocin-induced diabetic rats and 12 non-diabetic rats. There were five treatment groups: C2 tool antibody 0.01 µg treatment group (n=12), C2 tool antibody 1 µg treatment group (n=12), C2 tool antibody 50 µg treatment group (n=12), diabetic control group (n=12) and non-diabetic control group (n=12). At Week 3 post-hyperglycaemia induction, rats in the C2 tool antibody treatment groups received a single intravitreal injection of C2 tool antibody (0.01, 1

or 50 µg/eye in 4.7 µL), and rats in the two control groups received a single intravitreal dose of the anti-TNP antibody (4.5 µL; 11.2 mg/mL).

In all three experiments conducted on streptozotocin-induced diabetic rats, retinal function was assessed via electroretinography (ERG) recordings at baseline and at the end of each study (Weeks 7–9). During the neuroprotection experiment, ERG measurements were also obtained throughout the study period. All hyperglycaemic rats were randomised into treatment groups based on baseline ERG results to ensure that rats in all groups had a similar distribution of retinal dysfunction. After the final ERG recording, animals were sacrificed by overdose of intraperitoneal pentobarbital. Intravitreal injections were performed under a dissecting microscope using a 34-gauge needle fitted on a 10 µL Hamilton glass syringe. The general quality of injection was controlled by fundoscopy (Micron IV Retinal Imaging Microscope; Phoenix Research Labs, CA, USA). Prior to all intravitreal injections, streptozotocin-induced diabetic rats were anaesthetised with 2.5–3% isoflurane (AbbVie, North Chicago, IL, USA) and administered a drop of oxybuprocaine hydrochloride (4 mg/mL; OmniVision, Santa Clara, CA, USA).

The effects of the C2 tool antibody on restoration of retinal function were evaluated in the oxygen-induced retinopathy (OIR) mouse model

The effect of the C2 tool antibody on retinal function restoration was investigated in 26 mice with OIR and 15 normoxic control mice. There were three treatment groups: ischemic C2 tool antibody treatment group (n=31 eyes), ischemic control group (n=13 eyes) and normoxic control group (n=25 eyes). Pregnant mice (C57BL/6J) were obtained from Janvier Labs (Le Genest-Saint-Isle, France). The development of a central avascular retinal region was induced by exposure of the pups to 75% O_2 between postnatal day (P)7 and P12. This region became ischemic at P12 when the pups were exposed to room air. Starting from P12, the avascular area started to revascularise; however, ischemia-induced neurodegeneration and retinal function persisted.

At weaning (P21), pups were transferred to individually ventilated cages, maintained at a controlled temperature and humidity, with a 12-hour light/dark cycle and *ad libitum* access to food and water. The pups were allowed to acclimatise to this new environment for 1 week before the start of the study. After the development of ischemia, mice in the ischemic C2 tool antibody treatment group received two intravitreal injections of C2 tool antibody (1 µL; 11 mg/mL), administered 21 days apart. Normoxic and ischemic control mice received intravitreal injections of anti-TNP (1 µL; 11.6 mg/mL) administered as two doses, 21 days apart.

Prior to all intravitreal injections, OIR-induced ischemic mice were anaesthetised with 2.5–3% isoflurane (AbbVie) and administered a drop of oxybuprocaine hydrochloride (4 mg/mL; OmniVision) for additional topical, local anaesthesia. Intravitreal injections were performed under a dissecting microscope using a 34-gauge needle fitted on a 10 µL Hamilton glass syringe. The general quality of injection was controlled by fundoscopy and optical coherence tomography (OCT) using Micron IV Retinal Imaging Microscope (Phoenix Research Labs). Data were omitted if intravitreal injection-related injury to the retina was detected.

Retinal function was assessed via ERG recordings, and retinal morphology was evaluated by OCT; assessments were performed at baseline and after administration of each intravitreal injection. OCT images were taken after the ERG recordings. After the final ERG recording or OCT image, animals were sacrificed by an overdose of intraperitoneal pentobarbital. To assess retinal function, changes from P7 to P82 were investigated in rod-, UV-cone- and M-cone-driven b-wave light sensitivities; UV-cone- and M-cone-driven maximal oscillatory potentials; and cone-driven pattern ERG responses. At study termination, retinas were extracted and prepared for immunohistochemistry.

Safety of intravenous BI 754132 in cynomolgus monkeys

A total of 32 sexually mature cynomolgus monkeys (Covance Research Products, Denver, PA, USA) were housed in pairs in stainless steel cages maintained at controlled temperature and humidity conditions (20–26°C; 30–70% humidity; ≥8 air changes/hour), with a 12-hour light/dark cycle for at

least 3 weeks before the start of the study and throughout the study period. The monkeys were fed one or two times a day (Certified Primate Diet 5048; PMI Nutrition International, Richmond, IN, USA) and had *ad libitum* access to water. Food and water samples were routinely analysed for environmental contaminants. Animals were provided with various cage-enrichment devices and dietary enrichment, and were allowed to socialise under supervision.

At study start, the monkeys were randomised into four groups to receive intravenous BI 754132 (3 mg/kg, n=6; 10 mg/kg, n=6; 50 mg/kg, n=10) or vehicle control (n=10) once weekly for at least 13 weeks. The resultant dose groups were balanced with respect to bodyweight and sex. At the end of the treatment period (Week 13), six animals (three males and three females) in each group were euthanised; the remaining eight animals (BI 754132 50 mg/kg group, n=4; vehicle, n=4; four males and four females) were followed for another 13 weeks. Blood and urine samples were collected at baseline, on Days 28 and 91 of the treatment period, and on Days 85 and 91 of the follow-up period.

Safety of intravitreal BI 754132 in cynomolgus monkeys (13-week study)

A total of thirty-two 24–50-month-old cynomolgus monkeys (Charles River Laboratories) were housed in groups of up to three in stainless steel cages maintained at controlled temperature and humidity conditions (20–26°C; 30–70% humidity; ≥8 air changes/hour), with a 12-hour light/dark cycle for at least 3 weeks before the start of the study and throughout the study period. The monkeys were fed one or two times a day (Certified Primate Diet 5048) and had *ad libitum* access to water. Food and water samples were routinely analysed for environmental contaminants. Animals were provided with various cage-enrichment devices and dietary enrichment, and were allowed to socialise under supervision.

At study start, the monkeys were randomised into four groups to receive intravitreal BI 754132 (1 mg/eye, n=6; 3 mg/eye, n=6; 6 mg/eye, n=10) or vehicle control (n=10) for 13 weeks. The resultant dose groups were balanced with respect to bodyweight and sex. Monkeys randomised to BI 754132 1–3 mg/eye received one intravitreal injection every 4 weeks (50 µL; right eye); whereas those

randomised to BI 754132 6 mg/eye or vehicle control received two 50 µL injections every 4 weeks (both right eye), spaced at least 10 minutes apart. At the end of the treatment period (Week 13), six animals (three males and three females) in each group were euthanised; the remaining eight animals (BI 754132 6 mg/eye group, n=4; vehicle control, n=4; four males and four females) were followed for another 8 weeks.

Safety of intravitreal BI 754132 in cynomolgus monkeys (26-week study)

Forty 24- to 36-month-old cynomolgus monkeys (Bioculture US LLC, Immokalee, FL, USA) were housed in groups of up to three in stainless steel cages maintained at controlled temperature and humidity conditions (20–26°C; 30–70% humidity; ≥8 air changes/hour), with a 12-hour light/dark cycle and were acclimated to the test facility for 42 (males) or 49 (females) days prior to initiation. The monkeys were fed one or two times a day (Certified Primate Diet 5048) unless deprived of food for study procedures and had *ad libitum* access to water. Animals were provided with various cage-enrichment devices and dietary enrichment.

Male and female monkeys were assigned to four groups to receive intravitreal BI 754132 (1 mg, n=8; 3 mg, n=8; 6 mg, n=12) or vehicle control (n=12) to the right eye every 4 weeks. Monkeys assigned to BI 754132 1–3 mg/eye received one intravitreal injection every 4 weeks (50 µL); whereas those randomised to BI 754132 6 mg/eye or vehicle control received two 50 µL injections every 4 weeks, spaced at least 10 minutes apart. At the end of the treatment period (Week 26), eight animals (four males and four females) in each group were euthanised; the remaining eight animals (BI 754132 6 mg group, n=4; vehicle control, n=4; two males and two females per group) were followed for another 12 weeks.

Statistical Analysis of Preclinical Data

In the *in vitro* studies and the *in vivo* murine studies, statistical analyses were performed using one-way analysis of variance (ANOVA) followed by Tukey's multiple comparisons test (GraphPad Prism, Version 6.01). In some cases, an additional unpaired t-test was also performed. ERG data collected

during the *in vivo* murine studies were processed and analysed using the MATLAB software (version R2014a; MathWorks). In the two 13-week studies in cynomolgus monkeys, ANOVA (one way) and pairwise comparisons were used to analyse changes in blood pressure (intravenous study only), bodyweight, organ weight, electrocardiographic data (intravenous study only), immunophenotyping data (intravenous study only) and clinical pathology values. Prior to ANOVA, Levene's test was used to test for equality of variances between dose groups; if the test was significant ($P \leq 0.05$), a rank transformation was applied before ANOVA was conducted. If the ANOVA results were significant ($P \leq 0.05$), Dunnett's test was used for pairwise comparisons between each treated and control group. Data for each sex were analysed separately. Although descriptive data were collected during follow-up, due to the small sample size, no hypothesis testing was performed. In the study investigating the safety of intravitreal BI 754132, changes in intraocular pressure (IOP) and ERG parameters for each sex were analysed using repeated measures analysis of covariance.

Clinical Methodology

Randomisation and Masking

Both the single-rising–dose (SRD) and multiple-dose (MD) parts of the trial were open label and non-randomised; patients were assigned to escalating dose groups in the order of their enrolment, and all study investigators and participants were aware of the assigned treatment. However, in the electrocardiogram (ECG) laboratory, readers involved with QT interval measurements were masked with respect to the treatment, visit and demographic information collected. A sham injection was not used, as comparison between the study and fellow eyes was considered adequate to derive a tolerable dose.

Global Protocol Amendments

- Amendment 1 was issued as a result of authority feedback. Changes included specification of sentinel dosing and communication, specification of the minimum observation period for each participant, the size of the evaluable dose group prior to dose escalation, specification of

the criteria for dose-limiting events, the duration of male contraception, and revised trial termination criteria

- Global amendment 2 was issued as a result of authority feedback. The amendment specified the minimum immediate post-dose observation period and the specific assessments required for each participant
- Global amendment 3 was issued as a result of authority, coordinating investigator, and ECG vendor feedback. Changes included addition of an American Diabetes Association (ADA) sampling time at Visit 7, adjustment of the inclusion criteria based on patient participation in previous trials, and specification of various information related to the ECG assessments.
- Global amendment 4 was issued to clarify the content and design of the MD part of the trial including the addition of a flow chart and timetable for pharmacokinetics (PK), ADA sampling and ECG recording. These changes were made based on results from the SRD part of the trial, which indicated that only the highest dose tested was expected to provide sufficient eye exposure and efficacy
- Global amendment 5 added mandatory indirect ophthalmoscopy to slit lamp and repeated IOP assessments as well as post-dose fundus autofluorescence, spectral-domain optical coherence tomography (SD-OCT) and colour fundus photography in order to ensure that any post-dose changes in the treated eye would be promptly recognised. Additional safety information collected during the trial was integrated into the clinical trial protocol. Criteria for and communication of the sentinel dose process were clarified. Updates were also made to inclusion criteria and exclusion criteria for the MD part of the trial
- Global amendment 6 included a new time window for PK and ADA sampling and ECG measurement to improve these assessments and correct various small errors. In terms of changes to analyses planned in the protocol, the statistical analysis plan specified three additional analysis sets (entered set, enrolled set and dose-limiting event [DLE] set)

Tables

Table S1. *In Vitro* Affinity of the C2 Tool Antibody and BI 754132 for Human TrkB

Table S2. Effects of BDNF, the C2 Tool Antibody and BI 754132 on TrkB Phosphorylation and Intracellular Signalling in CHO Cells Overexpressing Human TrkB

[a]n=7; [b]n=10; [c]n=3; [d]n=2; [e]n=6; [f]n=4. Emax is given as % of the maximal effect induced by saturating doses of BDNF.
AKT1/2/3 = serine/threonine kinase AKT isoforms 1/2/3; BDNF = brain-derived neurotrophic factor; CHO = Chinese hamster ovary; EC50 = half maximal effective concentration; Emax = maximal inducible effect; ERK1/2 = extracellular signal-regulated kinase 1/2; IP1 = inositol-1-phosphate; SEM = standard error of the mean; TrkB = tropomyosin-related kinase receptor type B.

Table S3. Effects of BDNF, the C2 Tool Antibody, BI 754132, and BI 754118 on TrkB Phosphorylation and Intracellular Signalling in Human Neuronal SH-SY5Y Cells

[a]n=16; [b]n=14; [c]n=6; [d]n=11; [e]n=8; [f]n=7; [g]n=15; [h]n=10. Emax is given as % of the maximal effect induced by saturating doses of BDNF.
AKT1/2/3 = AKT serine/threonine kinase 1/2/3; BDNF = brain-derived neurotrophic factor; EC50 = half-maximal effective concentration; Emax = maximal inducible effect; ERK1/2 = extracellular signal-regulated kinase 1/2; SEM = standard error of the mean; TrkB = tropomyosin-related kinase receptor type B.

Table S4. Toxicokinetic Profile of Intravenous BI 754132 in Cynomolgus Monkeys

[a]AUC of BI 754132 was calculated over the time interval from 0 to 168 hours post-dose in the intravenous injection study and 0–672 hours post-dose in the intravitreal injection study; [b]Presented as median (range); [c]n=4; [d]n=5. AUC = area under the concentration–time curve; C_{max} = maximum serum concentration of BI 754132 after a single intravitreal dose; PK = pharmacokinetics; q4w = once every 4 weeks; qw = once weekly; SD = standard deviation; t_{max} = time from dosing to maximum serum concentration of BI 754132.

Table S5. Selection Criteria for the Clinical Trial of BI 754132

AMD = age-related macular degeneration; BCVA = best corrected visual acuity; ETDRS = Early Treatment Diabetic Retinopathy Study; GA = geographic atrophy; IMP = investigational medical product; IOP = intraocular pressure; MD = multiple dose; SD-OCT = spectral-domain optical coherence tomography; SRD = single-rising dose; YAG = yttrium aluminium garnet.

Figures

Figure S1. Summary of in vivo studies investigating the effects of TrkB agonism on retinal function restoration (A), preservation of retinal function (B), retinal neuroprotection in STZ-induced diabetic rats (C) and retinal function restoration in the OIR mouse mode (D). ERG= electroretinography; lgG1 = immunoglobulin G1; ivt = intravitreal; P = postnatal day; OIR = oxygen-induced retinopathy; s.c. = subcutaneous; STZ = streptozotocin; TrkB = tropomyosin--related kinase receptor type B.

Figure S2. Summary of in vivo studies investigating lhe safely of intravenous (A) and intravitreal (B and C) B1 754132 in cynomolgus monkeys. Vertical blue boxes denote the point of euthanasia. q4w = once every 4 weeks; qw = once weekly; R = randomisation.

Figure S3. Mean potency of BDNF, C2 tool antibody and Bl 754132 for TrkB in CHO cells overexpressing human and cynomolgus TrkB (A) and human neuronal SH-SY5Y cells (B). **P* < 0.05; ***P* < 0.01 (one-way ANOVA with Tukey's multiple comparisons test; the data obtained for the C2 tool antibody and Bl 754132 in human neuronal SH-SY5Y cells were additionally compared with the unpaired t-test as indicated). Error bars indicate SEM. ANOVA = analysis of variance; BDNF = brain-derived neurotrophic factor; CHO = Chinese hamster ovary; EC50 = half maximal effective concentration; ns = not significant; SEM = standard error of the mean; TrkB = tropomyosin-related kinase receptor type B.

Figure S4. Effects of TrkB agonism on the regulation of mRNA levels in human neuronal SH-SYSY cells. ***P* < 0.01; ****P* < 0.001 (one-way ANOVA with Tukey's multiple comparisons test). Error bars indicate SEM. ANOVA = analysis of variance; BDNF = brain-derived neurotrophic factor; CT= cycle threshold; lgG1 = immunoglobulin G1; mRNA= messenger ribonucleic acid; SEM = standard error of the mean; TrkB = tropomyosin-related kinase receptor type B.

Figure S5. Formation of β3-tubulin-positive dendrites and synaptic connections induced by BDNF, C2 tool antibody and Bl 754132 in human neuronal SH-SYSY cells. Error bars indicate SEM. BDNF = brain-derived neurotrophic factor; SEM = standard error of the mean.

Figure S6. Mean value at each study point in rod-driven (A) and UV-cone-driven (B) b-wave light sensitivity, rod-driven (C) and UV-cone-driven (D) saturating b-wave response amplitude, UV-cone-

driven photopic negative response (E) and outer retinal contrast sensitivity (F) in a preclinical study investigating the effects of TrkB agonism on retinal function presentation in streptozotocin-induced diabetic rats relative to non-diabetic control rats. *$P < 0.05$; **$P < 0.01$; ***$P < 0.001$ (one-way ANOVA with Tukey's multiple comparisons test; the data obtained for hyperglycaemlc control group at baseline and Week 8 were additionally compared with the paired t-test as indicated). Error bars indicate SEM. CS was determined by mixed rod- and cone-driven flicker ERG responses (at 12 Hz). ANOVA = analysis of variance; CS = temporal contrast sensitivity; CScontrol = temporal contrast sensitivity control; ERG = electroretinography; M = intravitreal; ns = not significant; R_{max} = saturating response amplitudes of rod-driven b-waves; $R_{max\ control}$ = R_{max} normalised to the mean saturating response amplitude of controls; S = light sensitivity; $S_{control}$ = light sensitivity normalised to the mean light sensitivities of controls; SME = standard error of the mean; TrkB = tropomyosin-related kinase receptor type B; UV = ultraviolet.

Figure S7. Mean value at each study point in rod-driven (A) and UV-cone-driven (B) b-wave light sensitivity, rod-driven (C) and M-cone-driven (D) saturating b-wave response amplitude, rod-driven (E), UV-cone-driven (F) and M-cone-driven (G) b-wave implicit times relative to the control group, the light sensitivity of the UV-cone-driven photopic negative response (H) and rod-driven a-wave responses (I) in a preclinical study investigating the effects of TrkB agonism on retinal function restoration in streptozotocin-induced diabetic rates relative to non-diabetic control rates. *$P < 0.05$; **$P < 0.01$; ***$P <0.001$ (one-way ANOVA with Tukey's multiple comparisons test; the data obtained for the C2 tool antibody treatment group at baseline and Week 7 were additionally compared with the paired t-test, as indicated). Error bars indicate SEM. ANOVA = analysis of variance; ivt = intravitreal; ns = not significant; R_{max} = saturating response amplitudes of rod-driven b-waves; $R_{control}$ = normalised to the mean response of the non-diabetic control group; $R_{max\ control}$ = R_{max} normalised to the mean saturating response amplitude of controls; S = light sensitivity, $S_{control}$ = light sensitivity normalised to the mean light sensitivities of controls; SEM = standard error of the mean; TrkB = tropomyosin-related kinase receptor type B; UV = ultraviolet.

Figure S8. Mean value at each study point in rod-driven (A), UV-cone-driven (B) and M-cone-driven (C) b-wave light sensitivity, UV-cone-driven (D), M-cone-driven b-wave maximal oscillatory potentials (E) and cone-driven pattern ERG responses (F) in a preclinical study investigating the effects of TrkB agonism on retinal function restoration in OIR-induced ischemic mice relative to normoxic control mice. *$P < 0.05$; **$P < 0.01$; ***$P < 0.001$ (one-way ANOVA with Tukey's multiple comparisons test; paired t-test was also performed in some cases, as indicated). Error bars indicate SEM. ANOVA = analysis of variance; ERG = electroretinography; ivt = intravitreal; ns= not significant; OIR = oxygen-induced retinopathy; $R_{control}$ = normalised to the mean response of the non-diabetic control group; R_{max}= saturating response amplitudes of rod-driven b-waves; R_{max} = $R_{control}$ normalised to the mean saturating response amplitude of controls; S = light sensitivity; $S_{control}$ = light sensitivity normalised to the mean light sensitivities of controls; SEM = standard error of the mean; TrkB = tropomyosin-related kinase receptor type B; UV = ultraviolet.

Figure S9. Number of DAPI-positive (A) and Brn3a-positive (B) cells and the ratio of Brn3a:DAPI positive cells (C) in retinal ganglion cell layer following intravitreal administration of C2 tool antibody or anti-TNP control antibody in OIR-induced ischemic mice relative to normoxic controls. *$P < 0.05$; **$P < 0.01$; ***$P < 0.001$ (one-way ANOVA with Tukey's multiple comparisons test; paired t-test was also performed in some cases, as indicated). Error bars indicate SEM. ANOVA = analysis of variance; DAPI = 4',6-diamidino-2-phenylindole; ns = not significant; OIR = oxygen-induced retinopathy; SEM = standard error of the mean; TNP = trinitrophenol.

Figure S10. Mean change from baseline in BCVA of the study (left) and fellow eyes (right) over time in the SRD (A) and MD (B) parts of the Phase I trial (TS). Error bars show SD. BL was defined as last measurement before the first administration of BI 754132. BCVA = best corrected visual acuity; BL = baseline; MD = multiple dose; q4w = administration 4 times weekly; SD = standard deviation; SRD = single rising dose; TS = treated set.

Fig S11. Mean change from baseline in GA area in the study (left) and fellow eyes (right) over time in the SRD (A) and MD (B) parts of the Phase I trial (TS). Error bars show SD. BL was defined as treatment Visit 2 (Day 1). If no BL value was available at Visit 2, BL was defined as the last measurement taken at screening (Visit 1; Day -3). GA = geographic atrophy; MD = multiple dose; Q4wk = administration 4 times weekly; SD = standard deviation; SRD = single rising dose; TS = treated set.

Figure S12. Mean change from baseline in CRT of the study (left) and fellow eyes (right) over time in the SRD (A) and MD (B) parts of the Phase I trial (TS). Error bars show SD. BL was defined as treatment Visit 2 (Day 1). If no BL value was available at Visit 2, BL was defined as the last measurement taken at screening (Visit 1; Day -3). BL = baseline; CRT, central retinal thickness; MD = multiple dose; q4w= administration 4 times weekly; SD = standard deviation; SRD = single rising dose; TS = treated set.

Figure S13. Changes from baseline in b-wave implicit time in response to different flash intensity in the SRD part of the Phase I trial (TS). Each line represents one subject. SRD = single rising dose; TS = treated set.

Figure S14. Changes from baseline in b-wave implicit time in response to different flash intensity in the MD part of the Phase I trial (TS). Each line represents one subject. MD = multiple dose; q4w= administration 4 times weekly; TS = treated set.
