# Supplementary material for: Tropomyosin-Related Kinase Receptor Type B Agonism in Geographic Atrophy—The Translational Challenges from Preclinical Data to a First-in-Human Trial
Source: Ophthalmol Sci. 2026 May 3;6(7):101216. doi: 10.1016/j.xops.2026.101216 (PMC13311265; doi:10.1016/j.xops.2026.101216)
Supplement: Study group members [file mmc21.docx]

**The 1418.01 Study Group Members**

Andrew Lotery,^1^ Sanjiv Banerjee,^2^ Tomas Burke,^3^ Nicholas Beare,^4^ Sergio Pagliarini,^5^ Brian B. Berger,^6^ Dennis Michael Marcus,^7^ William Zachery Bridges,^8^ David Stuart Boyer,^9^ Sunil S. Patel,^10^ John C. Randolph^11^

**The 1418.01 Study Group Member Affiliations**

^1^Southampton General Hospital, Southampton, UK

^2^University Hospital of Wales, Cardiff, UK

^3^Bristol Eye Hospital, Bristol, UK

^4^Royal Liverpool University Hospital, Liverpool, UK

^5^University Hospital Coventry, Coventry, UK

^6^Retina Research Center PLLC, Austin, TX, USA

^7^Southeast Retina Center PC, Augusta, GA, USA

^8^Western Carolina Retinal Associates PA, Asheville, NC, USA

^9^Retina-Vitreous Associates Medical Group, Tarzana, CA, USA

^10^Retina Research Institute of Texas, Abilene, TX, USA

^11^Center for Retina and Macular Disease, Orlando, FL, USA
